# Establishment of Human-Induced Pluripotent Stem Cell-Derived Neurons—A Promising In Vitro Model for a Molecular Study of Rabies Virus and Host Interaction

**DOI:** 10.3390/ijms222111986

**Published:** 2021-11-05

**Authors:** Thanathom Chailangkarn, Nathiphat Tanwattana, Thanakorn Jaemthaworn, Sira Sriswasdi, Nanchaya Wanasen, Sithichoke Tangphatsornruang, Kantinan Leetanasaksakul, Yuparat Jantraphakorn, Wanapinun Nawae, Penpicha Chankeeree, Porntippa Lekcharoensuk, Boonlert Lumlertdacha, Challika Kaewborisuth

**Affiliations:** 1 Virology and Cell Technology Research Team, National Center for Genetic Engineering and Biotechnology (BIOTEC), National Science and Technology Development Agency (NSTDA), Pathum Thani 12120, Thailand; Nanchaya.wan@biotec.or.th (N.W.); Yuparat.jan@gmail.com (Y.J.); 2 Interdisciplinary Program in Genetic Engineering and Bioinformatics, Graduate School, Kasetsart University, Bangkok 10900, Thailand; Nathiphat.tan@gmail.com; 3 Computational Molecular Biology Group, Chulalongkorn University, Pathum Wan, Bangkok 10330, Thailand; keng.tj707@gmail.com (T.J.); sira.sr@chula.ac.th (S.S.); 4 Research Affairs, Faculty of Medicine, Chulalongkorn University, Pathum Wan, Bangkok 10330, Thailand; 5 National Omics Center, National Science and Technology Development Agency (NSTDA), Pathum Thani 12120, Thailand; sithichoke.tan@nstda.or.th (S.T.); wanapinun.naw@nstda.or.th (W.N.); 6 Functional Proteomics Technology, Functional Ingredients and Food Innovation Research Group, National Center for Genetic Engineering and Biotechnology (BIOTEC), National Science and Technology Development Agency (NSTDA), Pathum Thani 12120, Thailand; kantinan.lee@biotec.or.th; 7 Department of Microbiology and Immunology, Faculty of Veterinary Medicine, Kasetsart University, Bangkok 10900, Thailand; Penpitchac56@nu.ac.th (P.C.); Fvetptn@ku.ac.th (P.L.); 8 Center for Advance Studies in Agriculture and Food, KU Institute Studies, Kasetsart University, Bangkok 10900, Thailand; 9 Queen Saovabha Memorial Institute, Thai Red Cross Society, WHO Collaborating Center for Research and Training Prophylaxis on Rabies, 1871 Rama 4 Road, Pathumwan, Bangkok 10330, Thailand; Qsmibld@yahoo.com

**Keywords:** human-induced pluripotent stem cells, in vitro model, neurons, rabies virus, proteomics analysis, virus–host interaction

## Abstract

Rabies is a deadly viral disease caused by the rabies virus (RABV), transmitted through a bite of an infected host, resulting in irreversible neurological symptoms and a 100% fatality rate in humans. Despite many aspects describing rabies neuropathogenesis, numerous hypotheses remain unanswered and concealed. Observations obtained from infected primary neurons or mouse brain samples are more relevant to human clinical rabies than permissive cell lines; however, limitations regarding the ethical issue and sample accessibility become a hurdle for discovering new insights into virus–host interplays. To better understand RABV pathogenesis in humans, we generated human-induced pluripotent stem cell (hiPSC)-derived neurons to offer the opportunity for an inimitable study of RABV infection at a molecular level in a pathologically relevant cell type. This study describes the characteristics and detailed proteomic changes of hiPSC-derived neurons in response to RABV infection using LC-MS/MS quantitative analysis. Gene ontology (GO) enrichment of differentially expressed proteins (DEPs) reveals temporal changes of proteins related to metabolic process, immune response, neurotransmitter transport/synaptic vesicle cycle, cytoskeleton organization, and cell stress response, demonstrating fundamental underlying mechanisms of neuropathogenesis in a time-course dependence. Lastly, we highlighted plausible functions of heat shock cognate protein 70 (HSC70 or HSPA8) that might play a pivotal role in regulating RABV replication and pathogenesis. Our findings acquired from this hiPSC-derived neuron platform help to define novel cellular mechanisms during RABV infection, which could be applicable to further studies to widen views of RABV-host interaction.

## 1. Introduction

Rabies is a lethal disease for humans caused by the rabies virus (RABV), which is transmitted through a bite of infected animals. At the periphery, RABV enters motor nerve endings at neuromuscular junctions via a nicotinic acetylcholine receptor [1]. The virus then axonally retrogrades to reach the central nervous system (CNS) and disseminates, causing adverse CNS signs and ultimately death [2,3,4]. Humans infected with RABV show neurological symptoms, including furiousness and paralysis, as the virus invades the CNS [5]. At these later stages, patients are predicted to have a 100% mortality rate. RABV promotes axon/dendrite degeneration and impairs neurotransmitter transport of the infected neurons [6,7], consequently debilitating neuronal functions leading to neurological symptoms and death [3,5,8]. Despite extensive studies on rabies pathogenesis, many aspects of how neural cells in the CNS respond to infection and their protective mechanisms are not clearly defined and, thus, need further investigations to improve therapeutic interventions [9,10,11]. To effectively control RABV infection and cure the disease, it is undeniable that fundamental research focusing on virus–host interactions in a precise disease model is one of the critical necessities for discovering potential vaccines and antiviral drugs.

The use of in vitro models, i.e., permissive cell lines, is convenient and straightforward, providing valuable information on RABV biology and cellular responses. For instance, BHK-21 and neuroblastoma cells are widely used to investigate host responses to RABV infection and viral neuroinvasiveness [12,13]. However, utilization of these immortalized cell lines might not truly reflect host responses of the CNS because their genetic makeup, such as a defect in the RIG-I pathway in BHK-21 cells [14] and a p53 mutation in a neuroblastoma cell line [15], are distinct from those of human neural cells. These aberrations may disguise neuron-specific antiviral responses and virus–host interactions found in the CNS.

In contrast, using primary neurons isolated from animal brains to mimic infection in vivo is more pathologically relevant and widely applied to examine diseases in the CNS [6]. However, this ex vivo platform faces ethical difficulties, provides cells with limited proliferative capacity, and may not represent adult human neurons’ biology. The use of human-induced pluripotent stem cells (hiPSC) provides an alternative solution to overcome these issues and, therefore, enables an ease-of-use model for studying virus–host interactions at cellular and molecular levels [16,17,18].

Generated by introducing four transcription factors into adult fibroblasts [19], hiPSCs have the potential to develop into many cell types. They have been applied in medical research as a tool for drug development and modeling diseases, such as autism spectrum disorder (ASD) and schizophrenia [20,21]. In addition, the application of hiPSC technology in virology was applicable for in vitro modeling of neurotropic virus infections [16,22]. Previously, hiPSC-derived neurons were proposed as a model for investigating biological responses to RABV infection, focusing on differentially expressed inflammatory cytokines during infection with different RABV strains [17].

Regarding the study of virus–host interaction, omics technologies, such as genomics, transcriptomics, proteomics, and metabolomics, provide global profiling for a deeper understanding of cell biology under various treatments. Each approach has strengths and limitations in applying biological data to answer specific research questions. The proteomics-based analysis is a powerful platform to predict and identify cellular mechanisms during RABV infection in permissive cell lines and infected human and animal brain tissues [23,24,25,26]. Despite many relevant protein profiles reported in RABV-infected BHK21 and neuroblastoma cell lines, the relevance of the identified proteins in these non-neuronal cell lines is obscure [27,28]. On the other hand, the proteomics studies of infected brain tissues would provide data on predominant biological responses in a specific region or a whole brain related to clinical symptoms [23]. However, they cannot reveal neuronal cell-specific responses due to the cellular heterogeneity of the samples. Proteome-wide identification and functional analysis of RABV infection specifically to human neuron cells remain elusive.

To extend our understanding of RABV and host interaction, we herein established hiPSC-derived neural progenitor cells (NPCs), astrocytes, and neurons. After cell-specific phenotype characterizations, we investigated the effects of RABV infection in hiPSC-derived neurons using a LC-MS/MS proteomics approach. A comprehensive analysis of proteomic data revealed remarkable cellular perturbations and significant differences in proteins and pathways at the early and late phases of infection. This study emphasizes the exploitation of the hiPSC-derived neurons to study RABV infection in vitro. The model accelerates the exploration of new/undefined factors associated with neuropathogenesis and neuroprotective mechanisms, which will pave the way to create more effective vaccines, enhance disease diagnostics, and establish therapeutic interventions against rabies.

## 2. Results

### 2.1. Generation and Validation of hiPSC-Derived NPCs, Neurons, and Astrocytes

#### 2.1.1. The Presence of Cell-Specific Markers Determined by Immunofluorescence Assay (IFA)

**Figure 1:**
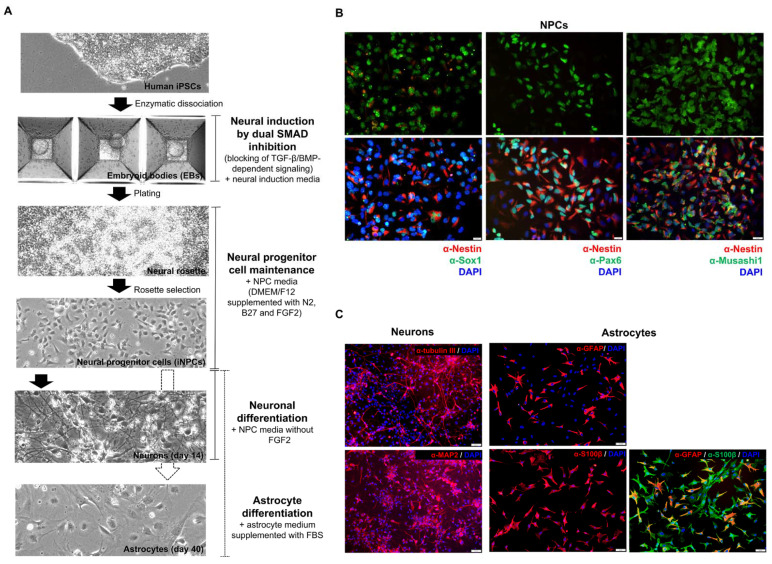
Generation and characterization of hiPSC-derived NPCs, neurons, and astrocytes and their expression of specific markers by IFA. (**A**) Schematic diagram summarizing the derivation of NPCs, neurons, and astrocytes from human iPSCs. (**B**) Undifferentiated NPCs were stained with mouse α-Nestin, rabbit α-Pax6, rabbit α-Musashi1, and rabbit α-Sox1 as primary antibodies. Goat α-mouse IgG Texas Red and goat α-rabbit IgG Alexa Fluor^®^ 488 were used as secondary antibodies. Scale bars = 20 µm. (**C**) Neurons were stained with mouse α-β-tubulin III and rabbit α-MAP2. Astrocytes were stained with mouse α-GFAP and rabbit α-S100β as primary antibodies. Goat α-mouse IgG Texas Red, α-rabbit IgG TRITC and α-rabbit IgG Alexa Fluor^®^ 488 were used as the secondary antibodies. Scale bars = 50 µm.

We first established an in vitro platform to obtain hiPSC-derived NPCs, progenitor cells for neurons and astrocytes. In this study, hiPSCs were derived and characterized from a healthy human donor [29]. The hiPSCs were used to generate NPCs via a dual-SMAD inhibition method (Figure 1A). Briefly, colonies of hiPSCs were enzymatically dissociated into single cells and aggregated, forming embryoid bodies (EBs). The EBs were fed with neural induction media to block the TGF-β/BMP-dependent SMAD signaling, resulting in cell differentiation toward the neural ectoderm lineage. The EBs were then plated into a new culture dish to create neural rosettes, which contain NPCs. Single NPCs growing out of the neural rosettes were maintained at an undifferentiated stage in NPC media comprised of DMEM/F12 plus N2, B27, GlutaMAX, antibiotic-antimycotic, and fibroblast growth factor (FGF2), which kept NPCs proliferating. To verify the characteristics of the derived NPCs, the expression of NPC-specific markers, including Nestin, Musashi1, Pax6, and Sox1, was confirmed by IFA (Figure 1B). Multipotency of NPCs was also demonstrated by their ability to differentiate into neurons and astrocytes. To generate neurons, NPCs were fed with NPC media without FGF2 (Figure 1A) every 3–4 days for at least 14 days. For astrocyte differentiation, NPCs were cultured in commercial astrocyte media containing 2% fetal bovine serum (FBS) (Figure 1A) for 30 days without passaging. Neurons, at 30 days post-differentiation (dpd) and astrocytes, at 40 dpd, exhibited distinctive morphologies and expressed their exclusive cell markers, β-tubulin III and MAP2 for neurons, and glial fibrillary acidic protein (GFAP) and S100β for astrocytes, respectively (Figure 1C).

#### 2.1.2. Transcriptomic Analysis of the hiPSC-Derived Cells

**Figure 2:**
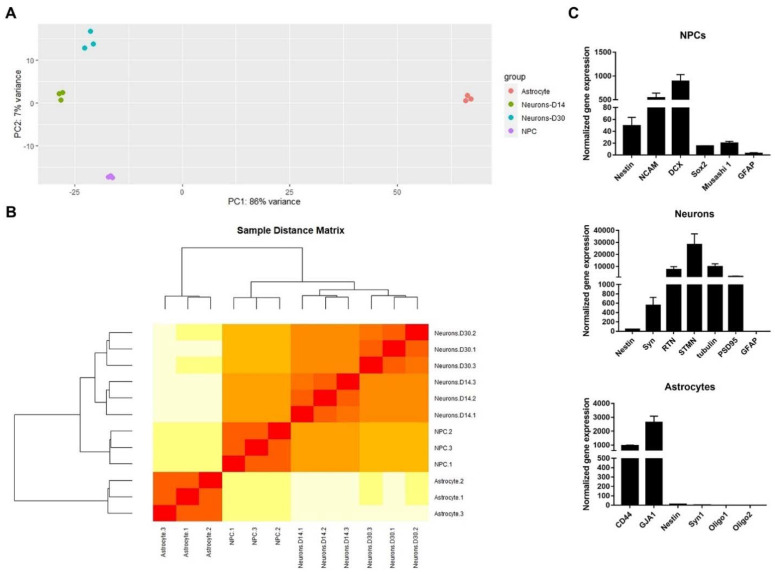
Transcriptomic and gene expression profiles of hiPSC-derived NPCs, neurons, and astrocytes. (**A**) Principal component analysis plot displaying all 12 RNA libraries along PC1 and PC2, which described 89% and 7% of the variability within the expression data set. (**B**) Hierarchical clustering analysis of all 12 RNA libraries based on Euclidian distances using DESeq2 package. (**C**) Gene expression profile of hiPSC-derived NPCs, neurons, and astrocytes quantified by real-time RT-PCR.

RNA sequencing was performed to examine the characteristics of the derived cells at the genomic level. RNA libraries were constructed from NPCs, neurons at 14 dpd (D14), neurons at 30 dpd (D30), and astrocytes, all of which were prepared in biological triplicate. All RNA libraries were sequenced and produced an average of 76.2 million reads per library (min: 43.3 million reads, max: 100.7 million reads), or 11.2 Gbp (min: 6.37 Gbp, max: 14.81 Gbp). The general profiles of each cell type were well separated when visualized using the first two components of a principal component analysis (PCA), with slight variations within three biological replicates (Figure 2A). This result was confirmed by hierarchical clustering analysis. The dendrogram demonstrated that neurons D14 and D30 had similar gene expression patterns (Figure 2B). The expression profiles of neurons were more identical to that of NPCs than that of astrocytes. We then quantified gene expression of specific genes in each cell type using RT-qPCR. Each of the derived cell types expressed higher levels of their stage-specific markers when compared to the others. Other cellular markers included neural cell adhesion molecule (NCAM), doublecortin (DCX), SOX2, and MSI1 for NPCs; Synapsin-1 (SYN1), Reticulon-1 (RTN1), Stathmin-2 (STMN2), and postsynaptic density-95 (PSD95) for neurons; and CD44 and GJA1 for astrocytes (Figure 2C). No expression of Olig1 and Olig2, markers of oligodendrocytes, was detected in the derived astrocytes (Figure 2C). Taken together, distinct populations of the derived neural cell types expressed their corresponding features and gene profiles.

### 2.2. hiPSC-Derived Neural Cells Allowed the Study of RABV Infection In Vitro

#### 2.2.1. RABV Permissiveness in Each hiPSC-Derived Cell Type

**Figure 3:**
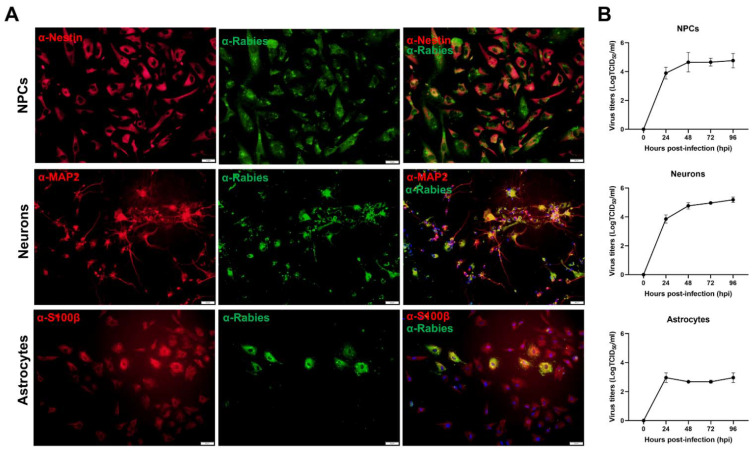
hiPSC-derived NPCs, neurons, and astrocytes are permissive to rabies infection. (**A**) All cell types were infected with RABV-TH at MOI of 0.5 for 72 h. The cultures were co-stained with horse α-rabies serum and mouse α-Nestin, MAP2, or S100β antibodies for NPCs, neurons, and astrocytes, respectively. The cells were observed by fluorescence microscopy. Scale bars = 50 µm. (**B**) Cells were infected with RABV-TH at MOI of 0.5. Cell supernatants were collected daily for virus titration in BHK21 cells. Error bars represent the standard deviation of virus titers (TCID_50_/_mL_) from three independent experiments.

We next investigated whether each derived cell type is permissive to RABV. The cells were infected with RABV-TH strain at an MOI of 0.5 for 72 h and subjected to an IFA using a horse α-RABV serum and antibodies against each cell type-specific marker. The presence of RABV proteins in the inclusion bodies and cytoplasm of infected cells suggests that NPCs, neurons, and astrocytes were all permissive to RABV infection (Figure 3A), though the replication was less efficient in astrocytes (Figure 3B). This seemingly abortive replication in astrocytes is in agreement with other in vitro and in vivo findings, which demonstrated a pivotal role of astrocytes in inhibiting RABV infection or spreading of the virus in the CNS through an activation of interferon-beta (IFN-β) and mitochondrial antiviral-signaling protein signaling pathway (MAVS) [30,31]. Our hiPSC-derived neurons were susceptible to RABV infection as reported by other groups [17], confirming that our system is applicable for in vitro molecular studies of RABV infection in the nervous system.

#### 2.2.2. RABV Induced Various Levels of Apoptosis Depending on hiPSC-Derived Cell Types

**Figure 4:**
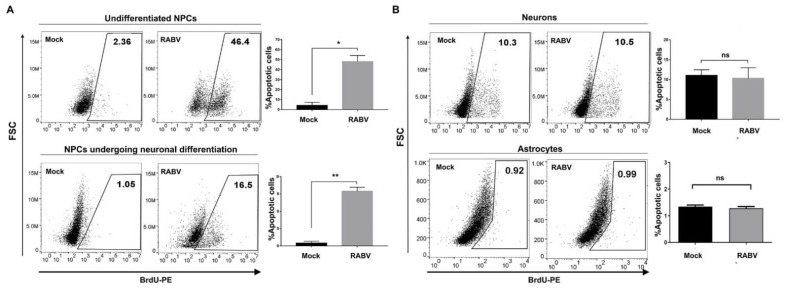
RABV infection induced apoptosis in hiPSC-derived NPC but not neurons and astrocytes. Cells were infected with RABV-TH at MOI 0.5. NPCs were maintained in NPC media (undifferentiated stage) or in NPC media with the withdrawal of FGF2 (NPCs undergoing neuronal differentiation). Neurons and astrocytes were maintained in NPC media without FGF2, and astrocyte media supplemented with FBS. To examine apoptosis in each cell type by flow cytometry, (**A**) NPCs at different stages included undifferentiated NPCs, and NPCs undergoing neuronal differentiation were harvested at 72 hpi and 5 days post infection (dpi), respectively, and (**B**) neurons and astrocytes were harvested at 72 hpi. Percentages of apoptotic cells shown are representative of triplicate. Error bars represent means ± SEM. Data were analyzed by an unpaired-*t* test. ns, *p* > 0.05; * *p* < 0.05; ** *p* < 0.01.

Apoptosis was implicated in RABV neuroinvasion and could be varied among RABV strains [32]. To examine whether RABV causes cell apoptosis in hiPSC-derived neural cells, we infected them with RABV-TH at an MOI of 0.5 and performed a TUNEL assay using flow cytometry. Like other neurotropic viruses reported to preferentially infect NPCs, RABV-TH significantly induced NPC apoptosis (Figure 4A). However, apoptosis was not observed in infected neurons and astrocytes (Figure 4B), correlating with previous reports describing a lack of apoptosis in hiPSC-derived neurons [17] and postmortem brains of humans infected with RABV [33]. These findings suggest that RABV modulates cell death at various degrees in different neural cell types.

NPCs located in parts of the cerebrum and hippocampus of adult brains are progenitor cells vitally responsible for reconstituting damaged neurons and glial cells. We hypothesized that RABV infection might hamper the differentiation of NPCs into neurons. To test this hypothesis, NPCs were either left untreated or infected with RABV-TH at an MOI of 0.5 and maintained in NPC media without FGF2 to induce neuronal differentiation. Cell supernatants were collected for 8 days and replenished with NPC media without FGF2 every 2 days after infection. In uninfected cultures, NPCs undergoing neuronal differentiation exhibited more neurons with elaborate structures, i.e., long and branching processes of axons and dendrites than those of infected cells (Figure S1A). RABV infectivity in NPCs was confirmed by a western blot and a virus titration. Increased viral protein expression and virus titers were detected in NPCs exposed to RABV, indicating that the virus efficiently replicated in NPCs undergoing neuronal differentiation (Figure S1B,C). Downregulated expression of the neuron markers, Nestin and β-tubulin III, was also observed (Figure S1D), suggesting that RABV altered NPC proliferation and neuronal differentiation. Nonetheless, whether RABV-infected NPCs consequently contribute to neuropathology in either rabies patients or animals remains unknown.

### 2.3. Proteomics Analysis of Infected hiPSC-Derived Neurons

#### 2.3.1. Temporal Changes in Protein Expression of RABV-Infected Neurons

**Figure 5:**
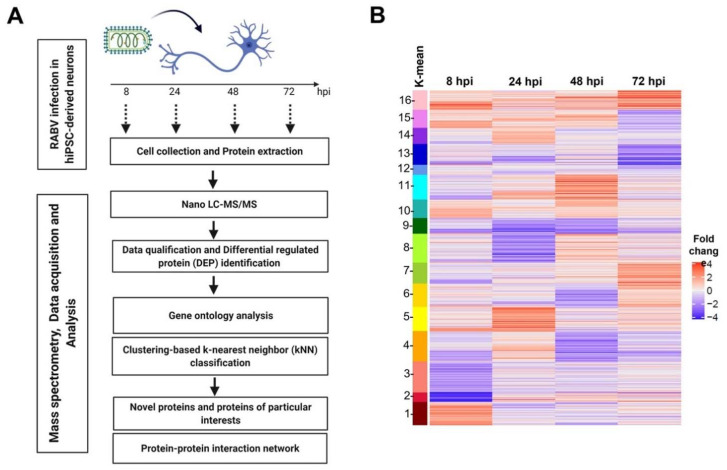
Schematic diagram of proteomic analysis and differential expression protein (DEP) identification in RABV-infected hiPSC-derived neurons. (**A**) Schematic workflows of sample preparation and bioinformatic analysis. (**B**) K-means clustering demonstrated 16 clusters of expressed proteins changing in RABV-infected neurons at 8, 24, 48, and 72 hpi.

Once the system for generating hiPSC-derived neurons was established, we focused our investigation on the effects of RABV infection on neuronal protein profiles. hiPSC-derived neurons were infected with RABV-TH at an MOI of 0.1. Cell lysates from infected and uninfected neurons were harvested at 8, 24, 48, and 72 hpi for protein identification using LC-MS/MS and bioinformatics analysis (Figure 5A). Differentially expressed proteins (DEPs) were detected with a fold-change cutoff of 1.5 and a z-score cutoff of 1.0 (see Materials and Methods). It should be noted that because our data contain two replicates, proteins with similar expression levels were pooled together to estimate the standard deviations of protein expression (Figure S2). A summary of identified proteins analyzed by LC-MS/MS in RABV-TH infected neurons compared to mock infection at each time point was shown by volcano plots and Venn diagrams (Figure S3). K-means clustering of DEP fold-change ratio performed by Genesis 1.8.1 software revealed temporal patterns of protein expression profiles during RABV-TH infection in neurons (Figure 5B and Supplementary File S1). Among the 3572 identified proteins, the expressions of 180, 171, 163, and 151 proteins were significantly downregulated, while 126, 151, 131, and 136 were significantly upregulated at 8, 24, 48, and 72 hpi, respectively (Supplementary File S2). Protein ID, average fold change, gene symbol, gene name, and Entrez gene of significant DEPs at each time point are described in Supplementary File S2.

We next validated our proteins found to be significantly down- or upregulated with those in previous studies of RABV-infected human and animal brain tissues (Table 1). Several proteins observed in our hiPSC-derived neuronal model were recognizably consistent with those found in infected brain samples, suggesting that our in vitro model is reliable and relevant for the RABV study.

#### 2.3.2. Gene Ontology (GO) Analysis of Proteins from Infected hiPSC-Derived Neurons

To obtain kinetic proteomic changes in infected neurons, we performed GO analysis at each time point. Shown by a bubble plot, enriched functions of DEPs in the GO database classified by biological process (Figure S4) and cellular component (Figure S5) were diverse across time points, demonstrating numerous host responses to RABV infection. We categorized them by their related cellular mechanisms, such as metabolic process, immune response, signal transduction, specific neuronal activity, cytoskeleton organization, and stress response (Tables S1–S6).

At 8 hpi, DEPs enriched in tumor necrosis factor (TNF) superfamily signaling pathway, cytokine production, and molecular mediator of immune response were downregulated. At the same time, carbohydrate-derivative catabolic and glycoprotein metabolic processes were upregulated. These suggest the interruption of host defense mechanisms and increased cellular energy metabolism at an early time point of virus infection. At 24 hpi, the proteins related to neurotransmitter, ion transport, and trans-synaptic transmission, presenting the largest group of enriched functions at this time point, were increased. At 48 hpi, the proteins associated with synaptic transport declined, along with proteins involved in the regulation of intermediate filament cytoskeleton and synaptic signaling. In contrast, those involved in Notch signaling and DNA damage response increased. Lastly, at 72 hpi, STAT cascade, proteins associated with microtubule organization, and positive regulation of cellular protein localization were downregulated. Some of the pathways of interest are elaborated below.

##### RABV Infection Changed Expressions of Proteins Involved in Immune Responses

As shown in Table S1, GO analysis revealed that RABV-TH infection of neurons, especially at an early time point (8 hpi), downregulated the expression of proteins involved in the innate and adaptive immune responses, including tumor necrosis factor superfamily cytokines (IFNG, BCL3, TICAM1), lymphocyte activation factors (IFNG, BCL3, RIF1), production of molecular mediator of immune responses (RIF1, TICAM1, cGAS, CARD11), those commonly expressed in response to viral infection (IFNG, BCL3, ADARB1, MST1R, ILF3, TICAM1, cGAS, POLR3B, NOP53), STAT cascades (IFNG, RET, NF2, PKD1, CTF1 and INPP5F), and signal transduction by p53 class mediators (BCL3, MAPKAPK5, DYRK2, TAF4B, TP63, NOP53) (Table S1). This downregulation of proteins involved in the immune response suggests the subversion of host defense mechanisms by RABV-TH at early time points of infection.

Among the proteins involved in the host immune responses, IFNG, is of great interest as it was categorized into several GOs (Table S1). IFNG is a proinflammatory cytokine responsible for innate and adaptive immune responses, such as antiviral stage activation, antigen processing and presentation enhancement, and lymphocyte trafficking. It is also one of the critical factors for activating type I IFN induction that is crucial for RABV clearance and reducing RABV pathogenicity in the CNS [36,37]. Due to this essential antiviral role of IFNG, viruses often evolve mechanisms to counteract IFNG functions. RABV was documented to suppress IFNG in mouse brains via suppressing DNA methylation and histone acetylation of the IFNG promoter [38]. Our in vitro model similarly identified that RABV-TH suppressed IFNG in infected neurons, suggesting that RABV-TH neuropathogenesis may involve the suppression of this important cytokine.

##### RABV Infection Changed Expression of Proteins Involved in Neurotransmitter Transport and Synaptic Vesicle Cycle

Neuronal dysfunction is implicated in RABV pathogenesis leading to deleterious outcomes mediated by neurotransmission impairment, ion-channel dysfunction, and neurotoxicity. Here, we drew attention to protein changes in the relevant biological processes, including neurotransmitter transport and synaptic vesicle. Through GO analyses, we classified the identified proteins into broad categories based on fundamental neuronal activity, including the neurotransmitter, synaptic transmission, synaptic vesicles, synaptic potential, glutamatergic synapse, and synaptic organization/assemble as shown in Table 2.

We found that expressions of proteins throughout the time course were disturbed with no recognizable patterns (Figure S4 and Table 2), suggesting various cellular compensatory responses to RABV infection. Following GO analysis of up- and downregulated proteins, we observed that the proteins could be classified into multiple groups of GO terms (Figure S6 and Supplementary File S3).

Expressions of multiple proteins, such as HSPA8, PCLO, ADORA2A, LRRK2, RHOA, TSPOAP1, and GRIN3A, enriched in the GO-glutamatergic synapse, were affected at different time points. We hypothesize that these proteins may play a role in neuropathogenesis during RABV infection. Previous studies showed that apart from their functions in mediating neurotransmitter and synaptic vesicle transport, ADORA2A [39], LRRK2 [40,41], SNCA [42], and TRPV1 [43] are also associated with the regulation of the neuroinflammatory response. A perturbation of ion homeostasis and synaptic activity resulting in neuronal dysfunction was also reported in RABV-infected mice. Moreover, upregulation of H^+^ ATPase and Na^+^/K^+^ ATPase and downregulation of Ca^2+^ ATPase can alter the intracellular Na^+^ and Ca^2+^ concentration, despite distinct responses to the pathogenic RABV strain SHBRV and the attenuated RABV strain B2C observed [34]. Altogether, our study and others suggest that RABV infection effectively modulates neurotransmission functions and inflammation of neuronal cells.

##### RABV Infection Changed Expression of Proteins Associated with Chaperone Systems

Molecular chaperones play essential roles in a cytoprotective response and cellular repair, mainly by regulating proteostasis (proper protein folding/refolding and assembly) under physiological and pathological conditions. Many viruses utilize this host machinery to modulate host cellular responses to achieve productive infection [44,45,46]. On the other hand, chaperones were also reported to be involved in antiviral defenses [47]. To date, only a limited number of studies described the association of chaperones with the RABV life cycle [48,49]. To broaden our understanding of the neuronal response to RABV infection, we aimed to delineate the protein changes associated with the chaperone systems.

**Figure 6:**
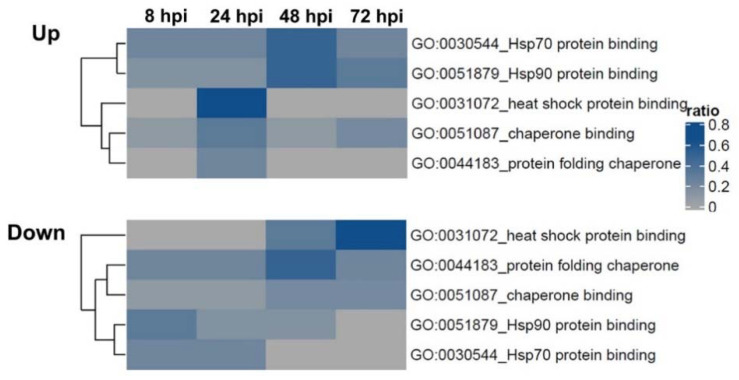
Hierarchical clustering of the proportion of DEPs among proteins annotated with each heat shock-related and chaperone function. Euclidean metric and complete linkage were used.

We found that RABV-TH regulated the expression of several chaperones and heat shock proteins (HSP), in particular, HSP90 and HSP70 (Figure 6 and Table 3). Moreover, we also observed changes in expression levels of HSP-associated proteins, such as HSPA8, ATP1A1, ATP1A2, and SNCA, which have crucial functions in regulating neurotransmitter and synaptic transmission.

Our results in Figure 6 highlight the changes in HSP90 during RABV infection. This may be a relevant pathway in virus–host interaction since HSP90 was shown to regulate the entry of several viruses, including enterovirus 71 virus and dengue virus [44,50]. Disruption of the HSP90 and viral RNA-dependent RNA polymerase (RdRp) complex also hampers viral protein expression and virus growth [51]. Together with our findings, HSP90 pathways may play an essential role in regulating RABV–host interactions.

We next performed a network analysis to illustrate the molecular interactions of the chaperones (Table 3) with other cellular proteins to identify their functionally relevant subnetworks using InnateDB (https://www.innatedb.com, accessed on 15 May 20121) and NetworkAnalyst databases (https://www.networkanalyst.ca, accessed on 18 June 2021) [52] to devise chaperone and HSP activities during RABV-TH infection. The results based on KEGG and GO analyses generated a map of proteins involved in protein folding, protein metabolic process, response to stress, virus infection, immune response, and regulation of programmed cell death. The map illustrates that HSPA8 and proteins in subnetworks, such as EIF2AK3, TLR4, NFKB1, BAK1, and RelA, are enriched in viral infection of several viruses (Figure S7A). The enrichment test also presents that the central nodes and their subnetworks, such as MAPK, chemokine, Toll-like receptor, and NOD-like receptor, are enriched among the proteins in the immune-signaling pathways (Figure S7B).

### 2.4. Protein–Protein Interaction Network of HSPA8

The central nodes identified from the network analysis (Figure S7) are likely to play a crucial role in the virus–host interaction. HSPA8 appeared to be a central node with the most elaborated networks connected to various functional interactions involving in glutamatergic synapse, neurotransmitter secretion (Table 2), virus life cycle, and immune-signaling pathways (Figure S7).

**Figure 7:**
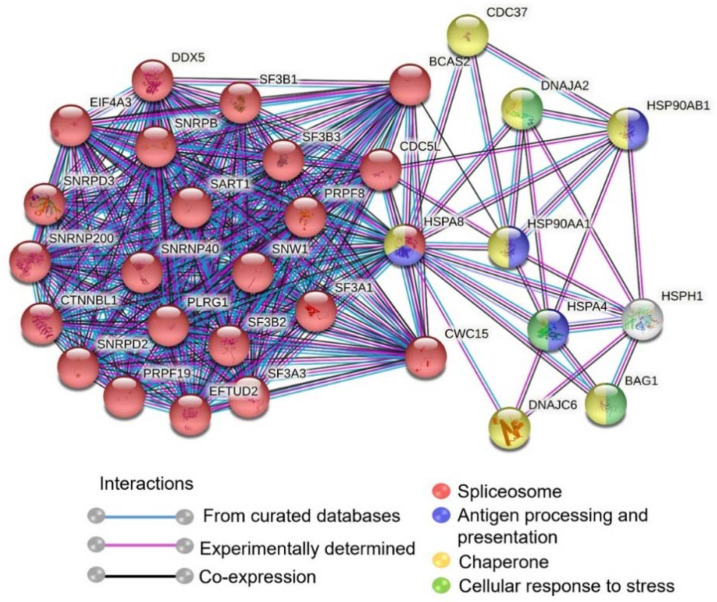
Protein–protein interaction network (PPI) of HSPA8 using STRING database. The edges represented in the full network indicate both functional and physical protein associations. Network statistics—number of nodes: 31, number of edges: 280, average node degree: 18.1, average local clustering coefficient: 0.933, expected number of edges: 73, PPI enrichment *p*-value: <1.0 × 10^−16^.

HSPA8 belongs to the HSP70 family, which is constitutively and abundantly expressed in neurons. The protein is essential for neuron recovery post-cellular stress [53]. We, thereby, performed protein–protein interaction networks of HSPA8 using the STRING database (http://string-db.org, accessed on 18 June 2021) [54] to assess pivotal molecular functions among the associated proteins (both direct and indirect association and co-expression). The results revealed that HSPA8 is related to spliceosome, chaperone, cellular response to stress, and antigen processing and presentation (Figure 7).

**Figure 8:**
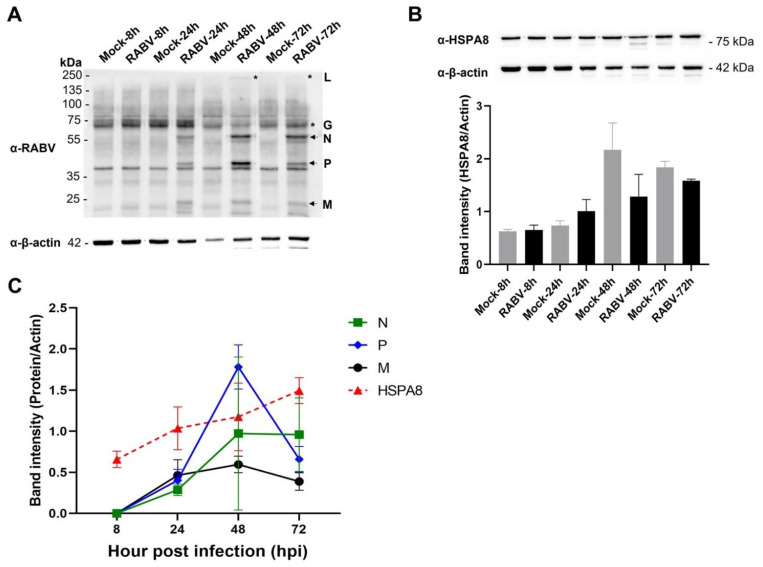
A dynamic change of RABV and HSPA8 proteins in infected hiPSC-derived neurons. Protein samples of mock and RABV-infected neurons were subjected to Western blot analysis. The membranes were probed with (**A**) horse α-RABV serum and (**B**) mouse α-human HSPA8 antibodies to detect RABV and HSPA8 proteins, respectively. β-actin was used as the internal loading control. (**C**) The intensity of RABV N, P, M, and HSPA8 in the infection group was normalized with β-actin using an image analysis program. * Note that RABV G and L proteins were excluded from the analysis due to nonspecific bands presented at the same size as RABV G, masking the RABV G and faint bands of RABV L. Error bars represent means of band intensity ± standard deviation of duplicates.

To illustrate the kinetics of the infection process and HSPA8 expression in infected neurons, we analyzed viral proteins in conjunction with HSPA8 at different time points (Figure 8A,B). Although we could not identify all rabies-specific peptides with high confidence due to the limited mass accuracy of our mass spectrometer (The peptide (LYDQVHIK) of the L protein was identified by Mascot with *p*-value < 0.05 (unadjusted *p*-value = 0.02)), the viral proteins were validated and measured by Western blot. The results showed that HSPA8 protein in infected neurons increased in correlation with the viral proteins, especially at 24–48 hpi (Figure 8C), suggesting that HSPA8 might respond to RABV-TH growth kinetics. This finding is consistent with a previous report demonstrating the dynamic expression of HSPA8 to regulate RABV replication via binding with RABV leader RNA (leRNA) [55].

### 2.5. Interaction of HSPA8 and RABV Proteins

**Figure 9:**
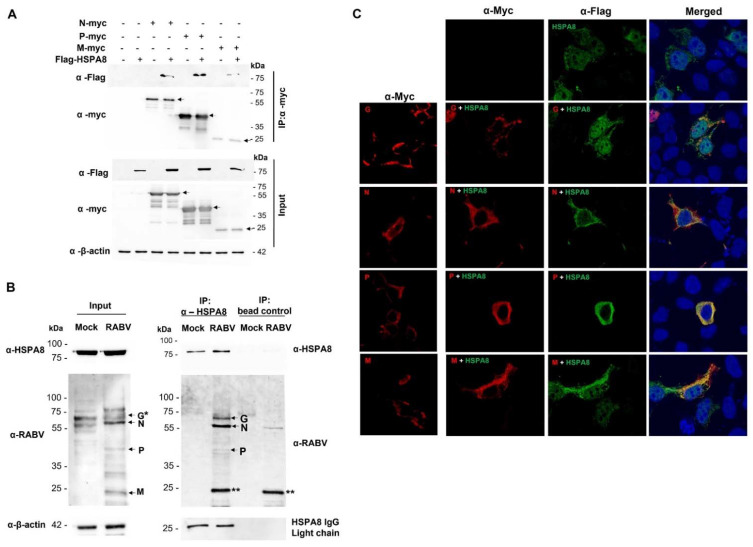
HSPA8 and RABV protein interactions. HSPA8 and RABV proteins’ physical interaction was determined by co-immunoprecipitations of protein samples harvested from HEK293T cells and hiPSC-derived neurons. (**A**) HEK293T cells were transfected with the plasmids expressing Flag-HSPA8 and RABV Nmyc, Pmyc, and Mmyc. At 48 h post-transfection, cell lysates were collected and prepared for immunoprecipitation using mouse α-myc beads. The eluted proteins were probed with rabbit α-myc and –flag. The arrows specify the presence of each viral protein according to its expected size (N, P, and M proteins at approximately 60, 40, and 25 kDa, respectively). (**B**) The neurons were infected with RABV-TH at MOI of 0.5. At 48 hpi, the cells were collected and subjected to immunoprecipitation using beads conjugated with mouse α-human HSPA8 antibody. The same samples were incubated with the beads without α-human HSPA8 antibody conjugation as the negative control. * RABV G appears at the same size as a nonspecific band found in a mock sample. ** RABV M might be either masked by the nonspecific band or strongly binds to the beads, despite lysate preclearing and several washing steps. (**C**) For the colocalization assay, transfected HEK293T cells were fixed with 4% paraformaldehyde and probed with mouse α-myc and rabbit anti-flag as primary antibodies. Goat α-rabbit Alexa Fluor 488 and goat α-mouse Alexa Fluor 647 were used as secondary antibodies.

Although there is a report presenting the effect of HSPA8 on RABV replication [55], the interaction between HSPA8 and RABV proteins and underlying mechanisms was not described. Here, we investigated the interaction between exogenous/endogenous HSPA8 and RABV proteins by performing co-immunoprecipitation assay. The results showed that exogenous HSPA8 physically interacts with the RABV N, P, and M in transfected HEK293 cells; however, with different degrees of binding affinity (Figure 9A). hiPSC-derived neurons were infected with RABV-TH at MOI of 0.5 for 48 h and subjected to immunoprecipitation to determine the binding of endogenous HSPA8 and RABV proteins. Endogenous HSPA8 could interact with RABV G, N, and P. However, we could not conclude whether endogenous HSPA8 is capable of binding with RABV M in infected neurons since the band at a similar size to RABV M appears in the eluted sample of the control beads (Figure 9B). A colocalization assay revealed that HSPA8 is typically localized in the cytoplasm and nucleus, but in the presence of RABV G protein, a majority of HSPA8 accumulated in the nucleus (Figure 9C). Conversely, the presence of HSPA8 reduced RABV N accumulation in the nucleus, while more abundance of RABV N was colocalized with HSPA8 at the perinuclear compartment (Figure 9C). Changes in the localization pattern of RABV M were also observed in the presence of HSPA8. These findings highlight the association of HSPA8 and RABV proteins that might play an essential role in regulating RABV proteins and ultimately regulate the virus life cycle.

## 3. Discussion

Neurons are the primary target of RABV infection. The virus axonally retrogrades from peripheral neurons to reach the CNS, causing detrimental symptoms and ultimately death [2,3,4]. Along with current human rabies therapeutic strategies, approaches for alleviating neuronal injury and regenerating new neurons were proposed for treating patients with a brain damage [9]. Such neuroregenerative approaches require an in-depth knowledge of the mechanisms underlying rabies neuropathogenesis. Reporting herein, we utilized hiPSC-derived neurons to investigate the effects of RABV infection on neuropathogenesis. We documented an alteration of cellular phenotypes, as well as changes in global protein profiles following a RABV infection. There were significant changes of proteins related to metabolic processes, immune and signal transduction, neurotransmitter transport, cytoskeleton organization, and cell stress response. We also drew attention to DEPs related to neuronal activities, immune response, and chaperone systems to delineate their significance on RABV pathogenesis and cellular defense against viral infection. Several proteins of great interest are highlighted below.

### 3.1. Novel Altered Proteins during RABV Infection and Their Significance

#### 3.1.1. Proteins Related to the Immune Response

Our study of RABV-infected, hiPSC-derived neurons identified proteins related to host immune responses, especially those involved in innate immunity. In particular, Toll/IL-1R homology domain-containing adaptor molecule 1 (TICAM-1), a protein associated with pattern recognition receptors (PRRs) signaling cascades, was found to be significantly downregulated during the RABV infection. TICAM-1 is a protein known to interact with the cytoplasmic tail of Toll-like receptor 3 (TLR3) and is reported to regulate antiviral activities in several virus infection models. For example, it was reported that TICAM-1 is essential for TLR3-induced type I IFN production responsible for a protective immune response against poliovirus. TICAM-1-deficient mice (*TICAM-1*^−/−^) exhibited a low level of type I IFNs in serum, spleen, and spinal cord and were more susceptible to poliovirus than the wild-type mice [56,57]. Conversely, TICAM-1 is dispensable for defending hepatitis B virus infection in *TICAM-1*^−/−^ mice [58]. Despite these reports, only scarce information on the roles of TICAM-1 in RABV infection is available. Many studies focused their investigations on TLR3 and found that TLR3 was upregulated in RABV-infected human NT2-N cells in correlation with an increased IFNβ production [59]. However, a differing study reported that TLR3 is dispensable for IFNβ production [60]. Additional exploration on the synergistic role of the TLR3/TICAM-1 pathways in modulating the immune response following RABV infection would provide more insights into their involvement in the virus replication and PRRs signaling cascades responsible for IFN-mediated antiviral activities.

Cyclic GMP-AMP synthase (cGAS) is another protein found in this study to be downregulated by RABV infection in the neurons. cGAS is a cytosolic DNA sensor accountable for an initiation of type I IFNs and inflammatory cytokine productions, primarily via cGAS–stimulator of interferon genes (STING) signaling [61,62]. Evidence of its inhibitory effects on RNA virus reproduction was described in vesicular stomatitis virus (VSV), a virus in the same family as RABV [63]. Therefore, we speculated that a reduction in cGAS expression after RABV infection might interfere with the cGAS–STING axis and type I IFN signaling, leading to efficient virus growth in RABV-TH-infected neurons.

The neuroinflammatory response is a common factor contributing to neuropathogenesis caused by viral infection. While it may lead to cell damage, neuroinflammation is crucial for viral clearance in the brain. Several cytokines involved are critical for immune cell infiltration, inflammation, and regulation of blood-brain barrier (BBB) permeability, all of which are processes that may alter neural functions and antiviral defense [64,65]. We found that one of these cytokines, IFNG, was modulated in RABV-infected neurons. IFNG is a proinflammatory cytokine responsible for innate and adaptive immune responses, including antiviral stage activation, antigen processing and presentation enhancement, and lymphocyte trafficking. However, excessive IFNG expression could also aggravate the disease, resulting in lethal outcomes in infected hosts. IFNG is one of the critical factors for activating type I IFNs needed for RABV clearance and reducing RABV pathogenicity in the CNS [36,37]. Expression of IFNG by a recombinant RABV enhances IFNG and early type I IFN production, leading to an inhibition of virus replication and spread in mouse brains and disease protection in a mouse model [66]. Importantly, upregulation of IFNG plays a pivotal role in combating both CVS and Nigerian street rabies virus (SRV) strains at early phases of infection. The downregulation of IFNG by RABV as found in this study may thus significantly contribute to the unimpeded viral replication leading to neuropathogenesis.

#### 3.1.2. Proteins Involved in the Neurotransmitter and Synaptic Transports

Neuronal dysfunctions, such as neurotransmission impairment, ion-channel dysfunction, and neurotoxicity, may contribute to RABV pathogenesis. Functional assessment of ion-channel activities, including membrane currents of voltage-dependent Na^+^ and Ca^2+^, delayed- and inward-rectifier K^+^, using whole-cell, patch-clamp recordings in mouse neuroblastoma cells, revealed the variability in functional alteration among the types of ion channels by RABV [67,68]. Inhibition or activation of such channels can affect neuronal action potential firing and neurotransmitter transport and lead to clinical behavioral symptoms and lethal disease outcomes [68]. In this study, we found that RABV induced changes in proteins enriched in the GO-glutamatergic synapse. These proteins include HSPA8, PCLO, ADORA2A, LRRK2, RHOA, TSPOAP1, and GRIN3A, which function in mediating neurotransmitter and synaptic vesicle transports [39]. Perturbation of these proteins may thus result in neuronal dysfunctions. It was reported in RABV-infected mice that an upregulation of H^+^ ATPase and Na^+^/K^+^ ATPase and downregulation of Ca^2+^ ATPase altered the intracellular Na^+^ and Ca^2+^ concentration [34]. A reduction of synaptic proteins, such as syntaxin-18, α-SNAP, and TRIM 9, also causes a pronounced accumulation of synaptic vesicles and an inhibition of synaptic vesicle release at the presynaptic membrane, leading to a severe neuronal dysfunction [34]. Together with our findings, it may be implied that rabies’ deleterious outcomes may be a result of RABV-mediated alterations of our discovered proteins related to synaptic activities.

Another protein involved in neurotransmitter transport found to be downregulated by RABV infection is synuclein alpha (α-Synuclein; SNCA). This protein is abundant in the brain and localizes to the synaptic terminals [69]. It was shown that a decreased expression of SNCA leads to a reduction in dopamine levels and transport [70]. Such impairment of neurotransmitter releases was shown to correlate with rabies disease progression [71]. In addition, an overexpression of SNCA protected SH-SY5Y neuroblastoma cells from oxidative stress [72], suggesting that SNCA is necessary for neuronal survival under pathological conditions. SNCA was also reported to upregulate the expression of IFN-stimulated genes (ISGs) in hiPSC-derived neurons following RNA virus infection and type I IFN treatment [73]. It was shown that an upregulation of SNCA suppressed viral replication in mouse brains and protected the mice from the virus infections (viruses in other viruses, including West Nile virus (WNV) and Venezuelan equine encephalitis virus (VEEV)) [73,74]. Our findings indicate that SNCA was suppressed in neurons after RABV infection, thus suggesting that RABV-mediated SNCA modulation may be involved in neuropathogenesis. Further investigations are needed to delineate the roles of SNCA in RABV growth and disease severity.

### 3.2. Potential Roles of Chaperones in Pathogenic and Neuroprotective Mechanisms

While it is known that HSPs exhibit a protective mechanism against cellular stress during viral infection, it is also evident that they promiscuously act as proviral factors supporting the survival of many viruses. For example, HSP90 is involved in the regulation of enterovirus 71 and dengue virus entry [44,50]. It also facilitates the replication of vesicular stomatitis virus (VSV) and other negative strand viruses by stabilizing viral proteins essential for the functions of RdRp [75,76]. HSP90 alpha (HSP90AA1), in cooperation with the Cdc37, helps maintain RABV P protein stability; however, it did not increase the amount of viral infectious particles [77]. Similar to HSP90, the HSP70 family of chaperones was reported to facilitate viral infection at multiple steps, including entry, replication, and assembly [51]. For RABV, HSP70 was found to be incorporated into RABV virions propagated in BHK-21 cells [78] and was shown to localize to the Negri bodies, where viral transcription and replication occur [79]. Moreover, a downregulation of HSP70 also suppresses RABV mRNA synthesis and virus particles [48], suggesting a pivotal role of HSP70 in RABV infection.

Our study highlighted HSPA8 as another chaperone protein actively involved multiple cellular pathways during RABV infection of the neurons. HSPA8 has various cellular functions, including mediation of clathrin dissociation from endosomes [80,81], induction of proper protein folding [82], ubiquitin-proteasome degradation pathway [83,84], and chaperone-mediated autophagy (CMA) [85]. The binding of HSPA8 to viral proteins also facilitated the replication of many viruses. For example, the interaction of HSPA8 with protein X of Bornavirus [86] or adenoviral hexon protein results in nuclear localization of proteins required for virus growth [86,87]. HSPA8 localized to the cell membrane interacts with the spike protein of the infectious bronchitis virus and facilitates virus infectivity [88]. These findings suggest the important roles of HSPA8 in multiple phases of the viral life cycle. Notably, our analyses suggest that HSPA8 can interact with the heat shock protein HSP90AA1 and the HSP90 co-chaperone or cell division cycle 37 (Cdc37) (Figure 7), which were demonstrated to maintain RABV P protein stability [77]. These data imply that the modulation of HSPA8 expression by RABV infection might enhance viral replication by promoting viral protein stability. While there are reports describing the binding of HSPA8 to RABV leader RNA [55], it is worth further examining the impact of HSPA8 on RABV in other aspects.

### 3.3. Strengths and Limitations of the Study

This study aimed to exploit the implementation of hiPSC-derived neurons, and other types of neural cells, for the study of RABV infection to examine molecular mechanisms of virus–host interaction in vitro. The strength of the study is that proteins associated with neuronal function were discovered. Various identified and selected proteins could potentially be used to assess their functions related to either neuroprotective response or viral neuropathogenesis.

The current study had a few limitations. First, in terms of the proteomics experimental design, a single RABV-TH strain was used due to our inaccessibility to other strains. Comparing the DEPs between different RABV strains would provide more intriguing and decisive data, since different strains could have distinct neuropathogenesis and elicit different neural responses. Other RABV strains, such as fixed or attenuated strains, could be included with the Thai strain in further studies. Second, evaluating the RABV life cycle in hiPSC-derived neurons that overexpressed or knocked down the protein of interest is considered a more relevant method for assessing the effects of identified host proteins. This approach was not utilized in this study due to our current lack of virus vector, particularly the adeno-associated virus (AAV) for gene delivery into hiPSC-derived neurons. Such a system is planned to be established in our laboratory. Further studies are warranted, with the hope that they will enhance knowledge of the field.

In summary, we established an hiPSC-derived neuron model for RABV infection and identified proteins related to metabolic processes, immune and signal transduction, neurotransmitter transport, cytoskeleton organization, and cell stress, to be infected by RABV-TH infection. We drew attention to DEPs related to neuronal activities, immune responses, and chaperone systems to deliberate their significance on RABV pathogenesis and cellular defense to virus infection. Our study provided more insights into the HSPA8 and RABV relationships, particularly physical interaction and colocalization of HSPA8 and RABV proteins. Further studies are warranted to identify underlying mechanisms associated with viral propagation and neuropathogenesis. Ultimately, our comprehensive proteomic analysis of RABV-infected hiPSC-derived neurons provides valuable information on the great potential of hiPSC-derived neurons as an in vitro elucidation of RABV-host interaction.

## 4. Materials and Methods

### 4.1. Cells and Virus

HiPSCs used in this study were kindly provided by Dr. Methichit Wattanapanitch (Siriraj Center for Regenerative Medicine (SiCRM), Research Department, Faculty of Medicine Siriraj Hospital, Mahidol University) [29]. The use of these iPSCs was approved by the Institutional Review Board (IRB) Ethic Committee of the National Science and Technology Development Agency (NSTDA) (NIRB-038-2562). HiPSCs were cultured on matrigel-coated plates and fed daily with mTeSR1 (STEMCELL Technologies, Vancouver, BC, Canada). At 70–80% confluence, cells were manually passaged. Briefly, hiPSC colonies were cut using a needle, washed once with PBS, covered with mTesR1, scraped off using a 1000-μL tip, and transferred to a new matrigel-coated plate.

HiPSCs, hiPSC-derived neural progenitor cells (NPCs), neurons, and BHK21.C13 cells (ATCC^®^) were maintained at 37 °C in a humidified atmosphere at 5% CO_2_.

The Thai-strain rabies virus (RABV-TH) was obtained from Queen Saovabha Memorial Institute (Bangkok, Thailand). The virus was isolated from a rabid dog’s brain and propagated in mouse brains for 7 passages. The virus was then subsequently cultured in BHK21.C13 cells for 5 passages to increase virus titers.

### 4.2. Derivation of Neural Progenitor Cells, Neurons, and Astrocytes

To generate NPCs, hiPSCs were enzymatically dissociated into single cells using the ACCUTASE™ cell detachment solution (STEMCELL Technologies), seeded onto AggreWell™800 24-well plates (STEMCELL Technologies) in STEMdiff™ Neural Induction Medium plus SMADi (STEMCELL Technologies). On the next day, cell aggregates, i.e., embryoid bodies (EBs), were visible, and three-fourths of the media in each well was subsequently changed daily. After 4 days, EBs were transferred to a matrigel-coated plate and fed daily with the same media. Four to six days after neural rosettes containing NPCs appeared in the culture, the rosettes were selectively detached from the culture plates using STEMdiff™ Neural Rosette Selection Reagent (STEMCELL Technologies) and plated onto a matrigel-coated dish in NPC media consisting of DMEM/F12 (HyClone, Logan, UT, USA), 0.5Χ N_2_ (Invitrogen, Waltham, MA, USA), 0.5X B27 (Invitrogen), 1X GlutaMAX (Gibco, Waltham, MA, USA), 1X antibiotic-antimycotic (Gibco), and 20 ng/mL fibroblast growth factor 2 (FGF2) (Sigma, St. Louis, MO, USA). Single NPCs growing out from replated rosettes (NPCs) were fed every other day with NPC media and enzymatically passaged using ACCUTASE™. Neuronal differentiation was initiated by the removal of FGF2 from the NPC media. The neuronal culture was fed every 3–4 days for at least 2–3 weeks. For astrocyte differentiation, NPCs were plated at a density of 15,000 cells/cm^2^ in NPC media. The next day, cells were fed with commercially available Astrocyte Medium (ScienCell, Carlsbad, CA, USA). Half of the media was changed every 3–4 days for 30 days without passaging. Astrocytes were then passaged using ACCUTASE™ and plated on a matrigel-coated plate.

### 4.3. RNA Sequencing

Total RNA samples were extracted from NPCs, neurons, and astrocytes using a Qiagen RNA Isolation Kit (Qiagen, Hilden, Germany) with DNAase I treatment (Qiagen). mRNA was isolated from total RNA using a Dynabeads mRNA Isolation Kit (Thermo Fisher Scientific, Waltham, MA, USA). mRNA samples (200 ng) were used to construct RNA libraries using MGIEasy RNA Library Prep kit (MGI, Shenzhen, China) and sequenced with MGISEQ-2000 sequencing platform. We used Salmon software [89] with default parameters to map RNA-seq reads to the human transcriptome release 37 from GENCODE (Frankish et al., 2019). The same software was used to count the number of RNA reads mapped to each reference transcript. The transcript-level quantification results were converted to the gene-level quantities with tximport [90] R package. Finally, DESeq2 [91] R package was used to normalize the gene-level quantities and plot PCA and heatmap from the normalization results.

### 4.4. Quantitative RT-PCR

Total RNA was isolated from NPCs, neurons, and astrocytes using Qiagen RNA isolation (Qiagen). Five hundred nanograms of total RNA were reverse transcribed to generate cDNA using a RevertAid RT Reverse Transcription Kit (Thermo Fisher Scientific). Forward and reverse primers (Table S7) were mixed with an equal amount of cDNA in an iTaq™ Universal SYBR^®^ Green Supermix (Bio-Rad, Hercules, CA, USA) following the manufacturer’s instructions. Each sample was prepared in biological triplicate and two technical replicates for quantitative real-time PCR (qPCR) using CFX96 Touch Real-Time PCR Detection System (Bio-Rad). The housekeeping gene, GAPDH, was used as an internal control for data normalization. The delta-delta Ct (ddCt) algorithm was used to calculate the relative change of gene expression [92].

### 4.5. Virus Infection

NPCs, neurons, and astrocytes were infected with RABV-TH at an MOI of 0.5 or otherwise indicated for 1 h. The cells were washed twice with phosphate-buffered saline (PBS). NPC media was used to maintain undifferentiated NPCs. Neurons and NPCs undergoing neuronal differentiation were maintained in NPC media without FGF2. Cell supernatants were collected at the indicated time points for viral growth curve analysis and subjected to virus titration.

For virus titration, BHK21 cells were grown in 96-well plates. Fifty microliters of ten-fold dilution of cell supernatant were transferred into each well of the 96-well plate in quadruplicate. The cells were incubated at 37 °C for 1 h_._ After incubation, the virus was removed. One hundred microliters of fresh Opti-MEM^®^ media (Gibco) were added to each well. At 72 h post-infection (hpi), cells were fixed with cold 80% acetone for 5 min and washed with PBS plus 0.05% Tween-20 (PBST). Horse anti-rabies serum (Queen Saovabha Memorial Institute) was added to each well to probe for rabies proteins. Dylight488-conjugated goat α-horse IgG (Abcam) was used as a secondary antibody. Virus titer (TCID_50_/_mL_) was calculated using the Reed and Muench method [93].

### 4.6. Immunocytofluorescence Assay

Cells grown in 6-well plates were fixed with 4% paraformaldehyde at 4 °C for 20 min. After fixation, cells were blocked and permeabilized with 3% bovine serum albumin (BSA) and 0.3% Triton-X for 30 min. The cells were then stained with primary antibodies diluted in 1% BSA for 1 h at room temperature. Primary antibodies used in the study included mouse α-Nestin (STEMCELL Technologies), rabbit α-Pax6 (STEMCELL Technologies), rabbit α-Sox1 (STEMCELL Technologies), rabbit α-Musashi1 (Abcam, Cambridge, UK), mouse α-β-tubulin III (STEMCELL Technologies), mouse α-GFAP (STEMCELL Technologies), mouse α-myc (Thermo Fisher Scientific), rabbit α-flag (Abcam), and horse α-rabies serum. After incubation, cells were washed three times with PBST, then stained with secondary antibodies. Secondary antibodies included goat α-mouse Texas Red (Abcam), goat α-rabbit TRITC (Abcam), goat α-rabbit Alexa Fluor^®^ 488, and goat α-horse IgG Dylight 488. After a washing step, nuclei were stained with VECTASHIELD^®^ Antifade Mounting Medium with DAPI (Vector Laboratories, Burlingame, CA, USA). The cells were observed by fluorescence microscopy (Olympus, Tokyo, Japan). For confocal microscopy, goat α-rabbit Alexa Fluor 488 and goat α-mouse 647 were used as secondary antibodies. Cells were mounted with ProLong™ Gold Antifade Mountant with DAPI (Invitrogen). The samples were observed by Fluoview™ FV1000 confocal microscopy (Olympus).

### 4.7. Western Blot Analysis

Cells were harvested using ACCUTASE™. Cell pellets were then lysed with mammalian lysis buffer (Thermo Fisher Scientific) containing 1Χ Halt™ protease inhibitor cocktail (Thermo Fisher Scientific). Cleared lysates were subjected to SDS-PAGE. Separated proteins on acrylamide gels were transferred to nitrocellulose membranes. The membranes were blocked with 5% skim milk for 1 h and probed with a primary antibody diluted in 5% skim milk for 1 h. Next, the membranes were washed three times with Tris-based saline and 0.1% Tween-20 (TBST) and probed with a secondary antibody diluted in 5% skim milk for 1 h. After the washing step, the protein bands were detected by adding chemiluminescence substrate (Bio-Rad) and visualized using ChemiDoc™ XRS+ imager (Bio-Rad).

### 4.8. TUNEL Assay by Flow Cytometry

NPCs, neurons, and astrocytes were infected with RABV-TH at an MOI of 0.5. Cells were harvested using ACCUTASE™ at the indicated time points. Cells were then subjected to the TUNEL assay using the in situ BrdU-Red DNA Fragmentation (TUNEL) Assay Kit (Abcam) according to the manufacturer’s protocols. Briefly, cells were fixed with 4% paraformaldehyde (*w/v*) for 15 min, followed by overnight fixation with ice-cold 70% ethanol (*v/v*). The fixed cells were washed, then treated with the DNA labeling solution containing TdT enzyme and BrdUTP at 37 °C for 60 min. After 2 washes with rinse buffer, cells were stained with an anti-BrdU antibody for 30 min in the dark at room temperature. Following a final wash, cells were analyzed for BrdU staining on a BD Accuri™ C6 Plus Flow Cytometer (BD Biosciences, Franklin Lakes, NJ, USA). Percentages of BrdU positive cells were calculated using the FlowJo version 9.9.6 program (FlowJo LLC, Ashland, OR, USA).

### 4.9. Nano-Liquid Chromatography and Tandem Mass-Spectrometry (Nano LC-MS/MS)

Neuron cells were lysed in 0.5% SDS lysis buffer and further vortex for 1 h at room temperature. The mixture was precipitated by centrifuged at 12,000 rpm for 30 min to discard the protein pellet. Total protein concentrations were quantified by the Lowry method [94]. At a sulfhydryl bond reduction step, 50 μg of each protein sample was incubated with 10 mM dithiothreitol (DTT) in 10 mM ammonium bicarbonate (NH_4_HCO_3_) at 56 °C. After incubation, 30 mM iodoacetamide (IAA) in 10 mM NH_4_HCO_3_ was added to block a reformation of the disulfide bond. The samples were then digested with 50 ng of sequencing-grade porcine trypsin (Promega, Walldorf, Germany) at 37 °C for 16 h. The tryptic peptides were dried, protonated in 0.1% formic acid (FA), and injected into an UltiMate 3000 Nano/Capillary LC System (Thermo Fisher Scientific, Sunnylvale, CA, USA) coupled to an HCTultra LC-MS system (Bruker Daltonics, Bremen, Germany) equipped with a nano-captive spray ion source. Briefly, peptides were enriched on a µ-Precolumn 300 µm i.d. X 5 mm C18 Pepmap 100, 5 µm, 100 A (Thermo Fisher Scientific), separated on an Acclaim PepMap™ 100 C18 column 50 mm internal diameter 0.075 mm, nanoViper (Thermo Fisher Scientific). Solvent A and B containing 0.1% formic acid in water and 0.1% formic acid in 80% acetonitrile were supplied on the analytical column. A gradient of 4–70% solvent B was used to elute the peptides at a constant flow rate of 0.30 μL/min for 30 min. Electrospray ionization was carried out at 1.6 kV using the CaptiveSpray. Mass spectra (MS) and MS/MS spectra were obtained in the positive-ion mode over the range (*m/z*) 400–1500 (Compass 1.9 software, Bruker Daltonics).

The MS/MS data was determined based on peptide MS signal intensities by the DeCyder MS differential analysis 2.0 software (GE Healthcare, Chicago, IL, USA). The PepDetect module was applied to evaluate and quantify the peptides with the following setups: mass resolution, 0.6; typical peak width, 0.1; ion trap mass resolution, 10,000; charge status, from 1 to 3; and *m/z* shift tolerance, 0.1 u. The PepMatch module was then used to measure signal intensity maps of each sample. Human proteome (Homo sapiens: 1,617,466 sequences; January 2021) was downloaded from the UniProt database and used in Mascot software (Matrix Science, London, UK) for human protein identification.

### 4.10. Differential Expression and Functional Enrichment Analyses of Proteomics Data

Three thousand five hundred seventy-two proteins that were consistently expressed in at least one replicate experiment of every condition were selected for further analysis. Assuming that zero expression values were due to the detection limit of LC-MS/MS technology rather than because the proteins were utterly absent in the cells, we calculated the average expression value for each condition by ignoring zeroes. To account for measurement biases, the expression profile of each condition was normalized against the reference condition (uninfected at an 8 h time point) of the same cell type. This was performed by calculating the log expression ratios between the condition of interest and the reference condition for all proteins, then scaling the expression values of the condition of interest so the median of log expression ratios is equal to 1.0. The main assumption was that if most proteins were unaffected by RABV infection, the log ratio of protein expression levels between any two conditions should center at 1.0.

To identify proteins with similar responses over time to RABV infection, the profile of log2 expression ratios between RABV-infected conditions and the corresponding uninfected control at the same time points (8 h, 24 h, 48 h, or 72 h) were calculated for each protein and subjected to a k-mean clustering analysis using Genesis 1.8.1 [95]. The number of the cluster was set at 16, which should be large enough to cover all significant patterns over the time points, and the number of iterations was set at 50.

Differentially expressed proteins (DEP) between a RABV-infected sample and the corresponding uninfected control at each time point were determined by applying a fold-change cutoff of 1.5 (0.58 log2FC) and a z-score cutoff of 1.0. The z-score of a protein was calculated as the ratio of (i) the difference between the average expression of infected and uninfected samples and the observed expression and (ii) the standard deviation of protein expression level in uninfected controls. As our data do not contain many replicates, proteins with similar expression levels were pooled to obtain robust estimates of standard deviations. Functional enrichment analyses of up- and downregulated DEPs at each time point were separately performed using the over-representation analysis method (ORA) of WebGestalt [96]. The set of 3572 proteins consistently observed across multiple experimental conditions was used as the background for statistical calculations. False discovery rates were estimated using the Benjamini–Hochberg technique. Top 20 enriched functions of each analysis were visualized and sorted by enrichment ratio. In addition, annotations of individual proteins were retrieved from the BioMart Ensembl database [97].

### 4.11. Co-Immunoprecipitation

HEK293T cells were cotransfected with the plasmids expressing Flag-HSPA8 and RABV proteins using FuGENE^®^ HD Transfection Reagent (Promega, Madison, WI, USA). At 48 h post-transfection (hpt), cell lysates were collected using pre-cooled IP Lysis Buffer (Pierce™, Thermo Fisher Scientific) supplemented with 1Χ Halt™ protease inhibitor cocktail (Thermo Fisher Scientific). Cleared lysates were incubated with mouse α-c-Myc Agarose (Pierce™, Thermo Fisher Scientific) with gentle rocking overnight at 4 °C. Immunoprecipitates were washed with TBST buffer (25 mM Tris-HCl, 0.15 M NaCl, 0.05% Tween-20, pH 7.2) and eluted in sample buffer, followed by SDS-PAGE and Western blot.

The hiPSC-derived neurons were infected with RABV-TH at MOI of 0.5. Mock infection was used as the negative control. At 48 hpi, the cells were collected and lysed by pre-cooled IP Lysis Buffer (Pierce™, Thermo Fisher Scientific) supplemented with 1Χ Halt™ protease inhibitor cocktail (Thermo Fisher Scientific). Forty-five micrograms of mouse α-human HSPA8 antibody were immobilized to 80 µL of AminoLink Plus Coupling Resin following the co-immunoprecipitation protocol described in the manufacturer’s instruction (Thermo Fisher Scientific). Each cleared lysate sample was divided into two parts for the incubation with the beads conjugated with mouse α-human HSPA8 antibody and bead control without antibody conjugation. After the incubation step, the protein complexes were eluted and analyzed by western blot. Horse α-RABV-TH serum was used to detect RABV proteins. Rabbit α-human HSPA8 (Abcam) and mouse α-human HSPA8 antibodies (Santa Cruz Biotechnology, Dallas, TX, USA) were used for the detection of HSPA8 protein and mouse HSPA8 IgG chains in the eluted samples.

### 4.12. Statistical Analysis

The differences in mean values between the two groups were analyzed by the unpaired *t*-test method using GraphPad Prism 5.0 (GraphPad Software Inc, San Diego, CA, USA). *p* values of <0.05 were considered statistically significant. All data are presented as means ± standard deviation (SD), unless stated otherwise.

## Figures and Tables

**Table 1:** Previously identified DEPs in RABV-infected brain samples and DEPs found in RABV-infected hiPSC-derived neurons.

Reported Protein in RABV-Infected Brain Samples			Relevant Proteins Found in RABV-Infected hiPSC-Derived Neurons						
Protein	Protein Change (Reference)	Sample	Gene Symbol	UniProt ID	GO:ID—Term Name	Protein Change			
						8 h	24 h	48 h	72 h
Na^+^/K^+^ ATPase	Upregulation [34]	SHBRV-infected mouse brain	ATP1A1 (ATPase Na^+^/K^+^ Transporting Sub-unit Alpha 1)	P05023	GO:0010644—cell communication by electrical coupling GO:0090662—ATP hydrolysis coupled transmembrane transport GO:0043209—myelin sheath	−0.03	**−3.09**	−1.55	0.30
			ATP1A2 (ATPase Na^+^/K^+^ Transporting Sub-unit Alpha 2)	P50993		1.12	**1.55**	0.85	**0.53**
Tubulin alpha-1C chain	Downregulation [35]	Human brain tissue infected by street rabies virus CVS-infected mouse brain	TUBA1C	Q9BQE3	GO:0006184—GTP catabolic process GO:0030705—cytoskeleton-dependent intracellular transport GO:0006457—protein folding	0.55	**−1.07**	**−1.58**	**−2.65**
Alpha enolase	Upregulation [26]	Human brain tissue infected by street rabies virus	ENO1	A0A2R8 Y879	GO:0006096—glycolytic process GO:0045296—cadherin binding	−1.54	0.001	−0.75	**1.31**
Alpha synuclein	Downregulation [35]	SRV-infected mouse brain	SNCA	P37840	GO:0071867—response to monoamine GO:0030133—transport vesicle GO:0071872—cellular response to epinephrine stimulus GO:0034599—cellular response to oxidative stress	−1.17	**−1.54**	−1.08	1.26
	Upregulation [25]	Street rabies virus-infected mouse hippocampal synaptosomes							

**Bold** represent significant changes at each time point.

**Table 2:** Gene ontology of DEPs associated with neuronal activities.

GO: ID	Term Name	Gene Symbol	UniProt ID	Protein Change			
				8 h	24 h	48 h	72 h
***Synaptic transmission***							
GO:0007268	chemical synaptic transmission	HTR5A	P47898	**1.25**	0.09	−0.26	−0.04
		SV2C	Q496J9	**−5.21**	0.44	−0.32	**−1.12**
		HTR7	P34969	**−2.80**	−0.61	**1.88**	**−2.36**
		MYCBPAP	Q8TBZ2	**−1.33**	0.10	−0.46	−0.41
		P2RX3	P56373	−0.58	−0.51	**−3.41**	−0.26
		SNCA	P37840	−1.17	**−1.54**	−1.09	1.26
		HAP1	P54257	−0.72	1.03	**−1.39**	**1.32**
		KCNK3	O14649	**1.10**	−0.48	0.58	−0.50
		CEP89	Q96ST8	−0.84	−0.66	−0.41	**−1.02**
		SDCBP	O00560	**−2.85**	0.31	0.29	**−3.48**
		APBA1	Q02410	0.27	1.05	0.37	**−1.73**
GO:0007274	neuromuscular synaptic transmission	P2RX3	P56373	−0.58	−0.51	**−3.41**	−0.26
		ADARB1	P78563	**−1.31**	−1.32	**−3.11**	**−1.48**
GO:0007271	synaptic transmission, cholinergic	ADORA2A	P29274	1.24	0.71	**−3.92**	**1.95**
GO:0035249	synaptic transmission, glutamatergic	ADORA2A	P29274	1.24	0.71	**−3.92**	**1.95**
GO:0032230	positive regulation of synaptic transmission, GABAergic	HAP1	P54257	−0.72	1.03	**−1.39**	**1.32**
GO:0051966	regulation of synaptic transmission, glutamatergic	ATP1A2	P50993	1.13	**1.56**	0.86	0.54
		LRRK2	Q5S007	−0.59	**1.51**	0.74	1.14
***Synaptic vesicle***							
GO:0016079	synaptic vesicle exocytosis	PCLO	Q9Y6V0	0.64	**1.27**	−0.46	0.51
		SNCA	P37840	−1.17	**−1.54**	−1.09	1.26
GO:0016189	synaptic vesicle to endosome fusion	EEA1	Q15075	−0.64	−0.15	**−1.53**	−1.51
GO:0036465	synaptic vesicle recycling	PLD2	O14939	−0.14	**1.55**	−0.08	1.21
		STON2	Q8WXE9	−0.12	1.21	**2.47**	0.17
GO:0048488	synaptic vesicle endocytosis	KIAA1109	Q2LD37	−0.64	−0.15	**−1.53**	**−1.51**
		SNCA	P37840	−1.17	**−1.54**	−1.09	1.26
		STON2	Q8WXE9	−0.12	1.21	**2.47**	0.17
GO:1900242	regulation of synaptic vesicle endocytosis	PACSIN1	Q9BY11	−1.15	**1.61**	−0.24	0.71
		LRRK2	Q5S007	−0.59	**1.51**	0.74	1.14
GO:2000300	regulation of synaptic vesicle exocytosis	ADORA2A	P29274	1.24	0.71	**−3.92**	**1.95**
		LRRK2	Q5S007	−0.59	**1.51**	0.74	1.14
		APBA1	Q02410	0.27	1.05	0.37	**−1.73**
		GRIN3A	Q8TCU5	1.15	**1.87**	1.34	−0.28
GO:2000807	regulation of synaptic vesicle clustering	PCDH17	O14917	1.00	0.00	**−2.35**	**−2.95**
***Synaptic potential***							
GO:0060079	excitatory postsynaptic potential	P2RX3	P56373	−0.58	−0.51	**−3.41**	−0.26
		ADORA2A	P29274	1.24	0.71	**−3.92**	**1.95**
		TRPV1	Q8NER1	**−1.38**	0.22	**−1.57**	**2.28**
		LRRK2	Q5S007	−0.59	**1.51**	0.74	1.14
GO:0090394	negative regulation of excitatory postsynaptic potential	LRRK2	Q5S007	−0.59	**1.51**	0.74	1.14
GO:0060078	regulation of postsynaptic membrane potential	GRIN3A	Q8TCU5	1.15	**1.87**	1.34	−0.28
***Presynapse***							
GO:1905606	regulation of presynapse assembly	SNCA	P37840	−1.17	**−1.54**	−1.09	1.26
GO:0099509	regulation of presynaptic cytosolic calcium ion concentration	TSPOAP1	O95153	**−1.65**	**−1.58**	1.24	−0.86
GO:1904071	presynaptic active zone assembly	PCLO	Q9Y6V0	0.64	**1.27**	−0.46	0.51
		PCDH17	O14917	1.00	0.00	**−2.35**	**−2.95**
***Glutamatergic synapse***							
GO:0098978	glutamatergic synapse	HSPA8	P11142	−0.83	**1.15**	**−1.46**	**−2.15**
		PCLO	Q9Y6V0	0.64	**1.27**	−0.46	0.51
		ADORA2A	P29274	1.24	0.71	**−3.92**	**1.95**
		LRRK2	Q5S007	−0.59	**1.51**	0.74	1.14
		RHOA	P61586	−0.13	**−1.79**	−0.56	−0.50
		TSPOAP1	O95153	**−1.65**	**−1.58**	1.24	−0.86
		HIP1	O00291	**−2.15**	**−3.99**	−0.76	−1.42
		PCDH17	O14917	1.00	0.00	**−2.35**	**−2.95**
		APBA1	Q02410	0.27	1.05	0.37	**−1.73**
		GRIN3A	Q8TCU5	1.15	**1.87**	1.34	−0.28
		SYNGAP1	Q96PV0	0.36	1.06	−0.05	**−2.54**
***Neurotransmitter***							
GO:0006836	neurotransmitter transport	SV2C	Q496J9	**−5.21**	0.44	−0.32	**−1.12**
GO:0098884	postsynaptic neurotransmitter receptor internalization	MX2	P20592	−0.84	0.36	**−1.18**	−0.49
GO:0046928	regulation of neurotransmitter secretion	MCTP1	Q6DN14	−0.99	**1.44**	**−1.98**	−0.96
GO:0007269	neurotransmitter secretion	HSPA8	P11142	−0.83	**1.15**	**−1.46**	**−2.15**
		TSPOAP1	O95153	**−1.65**	**−1.58**	1.24	−0.86
		APBA1	Q02410	0.27	1.05	0.37	**−1.73**
***Neuroinflammatory response***							
GO:0150076	neuroinflammatory response	IFNG	P01579	**−1.41**	−0.14	0.23	**−1.53**

**Bold** represent significant changes at each time point.

**Table 3:** Gene ontology of DEPs associated with chaperone system.

GO:ID	Term Name	Gene Symbol	UniProt ID	Protein Change			
				8 h	24 h	48 h	72 h
GO:0051087	chaperone binding	GET4	Q7L5D6	**−1.31**	0.22	−0.68	0.79
GO:0051879	HSP90 protein binding	TSC2	P49815	**−1.27**	−0.15	**−1.05**	−0.76
GO:0044183	protein folding chaperone	CCT6A	P40227	**−2.03**	−1.27	−0.58	−0.96
GO:0031072	heat shock protein binding	SSUH2	Q9Y2M2	−0.69	**1.91**	−0.91	**−1.54**
GO:0051087	chaperone binding	HSPA8	P11142	−0.83	**1.15**	**−1.46**	**−2.15**
GO:0044183	protein folding chaperone						
GO:0031072	heat shock protein binding						
GO:0051087	chaperone binding	ATP1A2	P50993	1.13	**1.56**	0.86	0.54
GO:0030544	HSP70 protein binding	SNCA	P37840	−1.17	**−1.54**	−1.09	1.26
GO:0044183	protein folding chaperone	CCT6B	Q92526	0.31	**−1.41**	**−1.34**	0.32
GO:0051879	HSP90 protein binding	NR3C1	P04150	0.23	**−1.23**	**1.20**	0.01
GO:0051087	chaperone binding	ATP1A1	P05023	−0.03	**−3.09**	**−1.55**	0.32
GO:0030544	HSP70 protein binding	ERN1	O75460	**−2.45**	0.65	**2.16**	0.44
GO:0051879	HSP90 protein binding						
GO:0051087	chaperone binding	LRP2	P98164	−0.07	−0.20	−0.39	**−1.27**
GO:0051087	chaperone binding	VWF	P04275	0.99	0.68	0.58	**1.39**
GO:0051879	HSP90 protein binding	EIF2AK3	Q9NZJ5	0.75	0.41	0.80	**2.00**
GO:0051879	HSP90 protein binding	STIP1	P31948	**1.28**	**1.94**	**2.09**	**1.85**
GO:0030544	HSP70 protein binding						
GO:0051087	chaperone binding						

**Bold** represent significant changes at each time point.

## Data Availability

The mass spectrometry proteomics data were deposited at the jPOST repository (https://repository.jpostdb.org/entry/JPST001315, Deposited on 13 September 2021).

## Supplementary Materials

The following are available online at https://www.mdpi.com/article/10.3390/ijms222111986/s1.
