# Supplementary material for: Establishment of Human-Induced Pluripotent Stem Cell-Derived Neurons—A Promising In Vitro Model for a Molecular Study of Rabies Virus and Host Interaction
Source: Int J Mol Sci. 2021 Nov 5;22(21):11986. doi: 10.3390/ijms222111986 (PMC8584829; doi:10.3390/ijms222111986)
Supplement: Supplementary file 1 [file ijms-22-11986-s001.zip › ijms-1414770-supplementary/IJMS-Supplementary Tables - submitted.pdf]

Table S1. Gene ontology of DEPs associated with host immune response and signal transduction

Time point (hpi)	GO	User ID	Gene symbol	Gene name	Fold change
8	GO:0071706 tumor necrosis factor superfamily cytokine production	P01579	IFNG	interferon gamma	-1.41
		P20749	BCL3	BCL3, transcription coactivator	-1.44
		Q8IUC6	TICAM1	toll like receptor adaptor molecule 1	-1.38
		Q9NUY8	TBC1D23	TBC1 domain family member 23	-1.35
	GO:0031929 TOR signaling	O43156	TTI1	TELO2 interacting protein 1	-4.00
		P49815	TSC2	TSC complex subunit 2	-1.27
		Q8IW41	MAPKAPK5	mitogen-activated protein kinase-activated protein kinase 5	-2.30
		Q8TEV9	SMCR8	Smith-Magenis syndrome chromosome region, candidate 8	-2.87
		Q9BXL7	CARD11	caspase recruitment domain family member 11	-1.10
	GO:0002285 lymphocyte activation involved in immune response	P01579	IFNG	interferon gamma	-1.41
		P20749	BCL3	BCL3, transcription coactivator	-1.44
		Q5UIP0	RIF1	replication timing regulatory factor 1	-2.53
		Q7KZ85	SUPT6H	SPT6 homolog, histone chaperone	-1.39
	GO:0002440 production of molecular mediator of immune response	Q5UIP0	RIF1	replication timing regulatory factor 1	-2.53
		Q7KZ85	SUPT6H	SPT6 homolog, histone chaperone	-1.39
		Q8IUC6	TICAM1	toll like receptor adaptor molecule 1	-1.38
		Q8N884	CGAS	cyclic GMP-AMP synthase	-2.99
		Q9BXL7	CARD11	caspase recruitment domain family member 11	-1.10
	GO:0002449 lymphocyte mediated immunity	P07437	TUBB	tubulin beta class I	-1.36
		P11226	MBL2	mannose binding lectin 2	-1.45
		P20749	BCL3	BCL3, transcription coactivator	-1.44
		Q5UIP0	RIF1	replication timing regulatory factor 1	-2.53
		Q7KZ85	SUPT6H	SPT6 homolog, histone chaperone	-1.39
	GO:0009615 response to virus	P01579	IFNG	interferon gamma	-1.41
		P20749	BCL3	BCL3, transcription coactivator	-1.44
		P78563	ADARB1	adenosine deaminase, RNA specific B1	-1.31
		Q04912	MST1R	macrophage stimulating 1 receptor	-3.80
		Q12906	ILF3	interleukin enhancer binding factor 3	-2.79
		Q8IUC6	TICAM1	toll like receptor adaptor molecule 1	-1.38
		Q8N884	CGAS	cyclic GMP-AMP synthase	-2.99
		Q9NW08	POLR3B	RNA polymerase III subunit B	-2.09
		Q9NZM5	NOP53	NOP53 ribosome biogenesis factor	-1.89

Table S1. Gene ontology of DEPs associated with host immune response and signal transduction *(Continued)*

Time point (hpi)	GO	User ID	Gene symbol	Gene name	Fold change
8	GO:0072331 signal transduction by p53 class mediator	P20749	BCL3	BCL3, transcription coactivator	-1.44
		Q8IW41	MAPKAPK5	mitogen-activated protein kinase-activated protein kinase 5	-2.30
		Q92630	DYRK2	dual specificity tyrosine phosphorylation regulated kinase 2	-1.94
		Q92750	TAF4B	TATA-box binding protein associated factor 4b	-1.71
		Q9H3D4	TP63	tumor protein p63	-4.15
		Q9NZM5	NOP53	NOP53 ribosome biogenesis factor	-1.89
	GO:000225 adaptive immune response	P01579	IFNG	interferon gamma	-1.41
		P11226	MBL2	mannose binding lectin 2	-1.45
		P20749	BCL3	BCL3, transcription coactivator	-1.44
		Q5UIP0	RIF1	replication timing regulatory factor 1	-2.53
		Q7KZ85	SUPT6H	SPT6 homolog, histone chaperone	-1.39
		Q8N1K5	THEMIS	thymocyte selection associated	-3.12
	GO:0038127 ERBB signaling pathway	O00443	PIK3C2A	phosphatidylinositol-4-phosphate 3-kinase catalytic subunit type 2 alpha	2.68
		Q6PJF5	RHBDF2	rhomboid 5 homolog 2	3.57
		Q8TBC3	SHKBP1	SH3KBP1 binding protein 1	2.02
48	GO:0070555 response to interleukin-1	P38159	RBMX	RNA binding motif protein X-linked	1.41
		Q92900	UPF1	UPF1, RNA helicase and ATPase	1.81
		Q9HAT8	PELI2	pellino E3 ubiquitin protein ligase family member 2	1.54
	GO:0007219 Notch signaling pathway	P46531	NOTCH1	notch receptor 1	1.06
		Q9BUL8	PDCD10	programmed cell death 10	1.54
		Q9NYJ7	DLL3	delta like canonical Notch ligand 3	1.29
		Q9Y4D7	PLXND1	plexin D1	2.99
72	GO:0097696 STAT cascade	P01579	IFNG	interferon gamma	-1.52
		P07949	RET	ret proto-oncogene	-2.18
		P35240	NF2	neurofibromin 2	-1.71
		P98161	PKD1	polycystin 1, transient receptor potential channel interacting	-2.02
		Q16619	CTF1	cardiotrophin 1	-1.98
		Q9Y2H2	INPP5F	inositol polyphosphate-5-phosphatase F	-2.53

Table S2. Gene ontology of differentially expressed proteins associated with metabolic processes

Time point (hpi)	GO	User ID	Gene symbol	Gene name	Fold change
8	GO:1901136 carbohydrate derivative catabolic process	Q08499	PDE4D	phosphodiesterase 4D	1.64
		Q6NSJ0	MYORG	myogenesis regulating glycosidase (putative)	1.39
		Q8N335	GPD1L	glycerol-3-phosphate dehydrogenase 1 like	1.50
		Q8WWQ2	HPSE2	heparanase 2 (inactive)	3.01
	GO:0006022 aminoglycan metabolic process	Q7LGC8	CHST3	carbohydrate sulfotransferase 3	3.32
		Q8IZ52	CHPF	chondroitin polymerizing factor	2.36
		Q8WWQ2	HPSE2	heparanase 2 (inactive)	3.01
	GO:0051117 ATPase binding	O75348	ATP6V1G1	ATPase H+ transporting V1 subunit G1	1.41
		Q08499	PDE4D	phosphodiesterase 4D	1.64
		Q7LBE3	SLC26A9	solute carrier family 26 member 9	2.28
	GO:0046434 organophosphate catabolic process	Q08499	PDE4D	phosphodiesterase 4D	1.64
		Q8IY17	PNPLA6	patatin like phospholipase domain containing 6	1.74
		Q8N335	GPD1L	glycerol-3-phosphate dehydrogenase 1 like	1.50
		Q9NY59	SMPD3	sphingomyelin phosphodiesterase 3	1.52
	GO:0009100 glycoprotein metabolic process	O60476	MAN1A2	mannosidase alpha class 1A member 2	3.19
		Q7LGC8	CHST3	carbohydrate sulfotransferase 3	3.32
		Q8IZ52	CHPF	chondroitin polymerizing factor	2.36
		Q8NBI6	XXYLT1	xyloside xylosyltransferase 1	1.68
		Q9NQ66	PLCB1	phospholipase C beta 1	1.29
		Q9NYU2	UGGT1	UDP-glucose glycoprotein glucosyltransferase 1	1.76
24	GO:0043112 receptor metabolic process	P37840	SNCA	synuclein alpha	-1.54
		Q68DV7	RNF43	ring finger protein 43	-1.41
		Q86UL8	MAGI2	membrane associated guanylate kinase, WW and PDZ domain containing 2	-1.64
		Q9Y2H2	INPP5F	inositol polyphosphate-5-phosphatase F	-1.27
	GO:0008202 steroid metabolic process	O95153	TSPOAP1	TSPO associated protein 1	-1.58
		P05023	ATP1A1	ATPase Na+/K+ transporting subunit alpha 1	-3.09
		P16234	PDGFRA	platelet derived growth factor receptor alpha	-1.44
		Q8WTV0	SCARB1	scavenger receptor class B member 1	-1.82
		Q9H4L5	OSBPL3	oxysterol binding protein like 3	-1.54

Table S2. Gene ontology of differentially expressed proteins associated with metabolic processes ***(Continued)***

Time point (hpi)	GO	User ID	Gene symbol	Gene name	Fold change
24	GO:0009141 nucleoside triphosphate metabolic process	O75081	CBFA2T3	CBFA2/RUNX1 translocation partner 3	-2.14
		P11055	MYH3	myosin heavy chain 3	-1.45
		P37840	SNCA	synuclein alpha	-1.54
		P61586	RHOA	ras homolog family member A	-1.79
		Q16795	NDUFA9	NADH:ubiquinone oxidoreductase subunit A9	-3.18
		Q9Y2B5	VPS9D1	VPS9 domain containing 1	-2.41
48	GO:0006644 phospholipid metabolic process	O00750	PIK3C2B	phosphatidylinositol-4-phosphate 3-kinase catalytic subunit type 2 beta	3.59
		O95427	PIGN	phosphatidylinositol glycan anchor biosynthesis class N	1.60
		P42356	PI4KA	phosphatidylinositol 4-kinase alpha	1.86
		Q6UWR7	ENPP6	ectonucleotide pyrophosphatase/phosphodiesterase 6	1.34
		Q8WTV0	SCARB1	scavenger receptor class B member 1	1.42
		Q96PC2	IP6K3	inositol hexakisphosphate kinase 3	1.85
		Q9BTU6	PI4K2A	phosphatidylinositol 4-kinase type 2 alpha	1.31
72	GO:0006766 vitamin metabolic process	P01579	IFNG	interferon gamma	-1.52
		P15328	FOLR1	folate receptor 1	-2.2
		P98164	LRP2	LDL receptor related protein 2	-1.27
	GO:0043112 receptor metabolic process	O00560	SDCBP	syndecan binding protein	-3.48
		Q86UL8	MAGI2	membrane associated guanylate kinase, WW and PDZ domain containing 2	-1.11
		Q9P2Y5	UVRAG	UV radiation resistance associated	-1.53
		Q9Y2H2	INPP5F	inositol polyphosphate-5-phosphatase F	-2.53

Table S3. Gene ontology of differentially expressed proteins associated with synaptic vesicle and neurotransmitter transports

Time point (hpi)	GO	User ID	Gene symbol	Gene name	Fold change
24	GO:0035249 synaptic transmission, glutamatergic	P21359	NF1	neurofibromin 1	1.53
		P50993	ATP1A2	ATPase Na+/K+ transporting subunit alpha 2	1.55
		Q5S007	LRRK2	leucine rich repeat kinase 2	1.50
	GO:0006836 neurotransmitter transport	P21359	NF1	neurofibromin 1	1.53
		P50993	ATP1A2	ATPase Na+/K+ transporting subunit alpha 2	1.55
		P78508	KCNJ10	potassium voltage-gated channel subfamily J member 10	2.95
		Q5S007	LRRK2	leucine rich repeat kinase 2	1.50
		Q6DN14	MCTP1	multiple C2 and transmembrane domain containing 1	1.43
		Q8TCU5	GRIN3A	glutamate ionotropic receptor NMDA type subunit 3A	1.86
		Q9Y6M5	SLC30A1	solute carrier family 30 member 1	1.25
		Q9Y6V0	PCLO	piccolo presynaptic cytomatrix protein	1.26
	GO:0006813 potassium ion transport	P50993	ATP1A2	ATPase Na+/K+ transporting subunit alpha 2	1.55
		P63165	SUMO1	small ubiquitin-like modifier 1	1.72
		P78508	KCNJ10	potassium voltage-gated channel subfamily J member 10	2.95
		Q92953	KCNB2	potassium voltage-gated channel subfamily B member 2	1.28
		Q9H4A3	WNK1	WNK lysine deficient protein kinase 1	2.18
	GO:0099504 synaptic vesicle cycle	O14939	PLD2	phospholipase D2	1.55
		Q5S007	LRRK2	leucine rich repeat kinase 2	1.50
		Q8TCU5	GRIN3A	glutamate ionotropic receptor NMDA type subunit 3A	1.86
		Q9BY11	PACSIN1	protein kinase C and casein kinase substrate in neurons 1	1.61
		Q9Y6V0	PCLO	piccolo presynaptic cytomatrix protein	1.26
	GO:0001505 regulation of neurotransmitter levels	P21359	NF1	neurofibromin 1	1.53
		P50993	ATP1A2	ATPase Na+/K+ transporting subunit alpha 2	1.55
		P78508	KCNJ10	potassium voltage-gated channel subfamily J member 10	2.95
		Q5S007	LRRK2	leucine rich repeat kinase 2	1.50
		Q6DN14	MCTP1	multiple C2 and transmembrane domain containing 1	1.43
		Q8TCU5	GRIN3A	glutamate ionotropic receptor NMDA type subunit 3A	1.86
		Q9Y6M5	SLC30A1	solute carrier family 30 member 1	1.25
		Q9Y6V0	PCLO	piccolo presynaptic cytomatrix protein	1.26

Table S3. Gene ontology of differentially expressed proteins associated with synaptic vesicle and neurotransmitter transports *(Continued)*

Time point (hpi)	GO	User ID	Gene symbol	Gene name	Fold change
24	GO:0099177 regulation of trans-synaptic signaling	P21359	NF1	neurofibromin 1	1.53
		P50993	ATP1A2	ATPase Na+/K+ transporting subunit alpha 2	1.55
		P78508	KCNJ10	potassium voltage-gated channel subfamily J member 10	2.95
		Q5S007	LRRK2	leucine rich repeat kinase 2	1.50
		Q6DN14	MCTP1	multiple C2 and transmembrane domain containing 1	1.43
		Q8TCU5	GRIN3A	glutamate ionotropic receptor NMDA type subunit 3A	1.86
		Q9Y6M5	SLC30A1	solute carrier family 30 member 1	1.25
	GO:0034765 regulation of ion transmembrane transport	P27105	STOM	stomatin	1.52
		P50993	ATP1A2	ATPase Na+/K+ transporting subunit alpha 2	1.55
		P63165	SUMO1	small ubiquitin-like modifier 1	1.72
		P78508	KCNJ10	potassium voltage-gated channel subfamily J member 10	2.95
		Q8TDI7	TMC2	transmembrane channel like 2	1.88
		Q92953	KCNB2	potassium voltage-gated channel subfamily B member 2	1.28
		Q9H4A3	WNK1	WNK lysine deficient protein kinase 1	2.18
		Q9Y6M5	SLC30A1	solute carrier family 30 member 1	1.25
	GO:0098793 presynapse	O14939	PLD2	phospholipase D2	1.55
		P11142	HSPA8	heat shock protein family A (Hsp70) member 8	1.15
		P21359	NF1	neurofibromin 1	1.53
		P78508	KCNJ10	potassium voltage-gated channel subfamily J member 10	2.95
		Q16849	PTPRN	protein tyrosine phosphatase, receptor type N	2.99
		Q5S007	LRRK2	leucine rich repeat kinase 2	1.50
		Q6DN14	MCTP1	multiple C2 and transmembrane domain containing 1	1.43
		Q99726	SLC30A3	solute carrier family 30 member 3	1.86
		Q9BY11	PACSIN1	protein kinase C and casein kinase substrate in neurons 1	1.61
		Q9Y6V0	PCLO	piccolo presynaptic cytomatrix protein	1.26
48	GO:0099504 synaptic vesicle cycle	O14917	PCDH17	protocadherin 17	-2.35
		P29274	ADORA2A	adenosine A2a receptor	-3.91
		Q15075	EEA1	early endosome antigen 1	-3.90
		Q86UL8	MAGI2	membrane associated guanylate kinase, WW and PDZ domain containing 2	-2.39

Table S3. Gene ontology of differentially expressed proteins associated with synaptic vesicle and neurotransmitter transports *(Continued)*

Time point (hpi)	GO	User ID	Gene symbol	Gene name	Fold change
48	GO:0051648 vesicle localization	O14917	PCDH17	protocadherin 17	-2.35
		P12259	F5	coagulation factor V	-2.07
		P15328	FOLR1	folate receptor 1	-2.35
		P29274	ADORA2A	adenosine A2a receptor	-3.91
		P54257	HAP1	huntingtin associated protein 1	-1.38
		Q86UL8	MAGI2	membrane associated guanylate kinase, WW and PDZ domain containing 2	-2.39
		Q9BZH6	WDR11	WD repeat domain 11	-1.94
	GO:0051271: negative regulation of cellular component movement	O94813	SLIT2	slit guidance ligand 2	-1.43
		P21359	NF1	neurofibromin 1	-1.55
		P35240	NF2	neurofibromin 2	-1.90
		P78563	ADARB1	adenosine deaminase, RNA specific B1	-3.11
		Q6DN14	MCTP1	multiple C2 and transmembrane domain containing 1	-1.97
		Q86SQ0	PHLDB2	pleckstrin homology like domain family B member 2	-1.57
		Q86UL8	MAGI2	membrane associated guanylate kinase, WW and PDZ domain containing 2	-2.39
		Q9NS66	GPR173	G protein-coupled receptor 173	-2.51
72	GO:0099177 regulation of trans-synaptic signaling	O14917	PCDH17	protocadherin 17	-2.35
		P21359	NF1	neurofibromin 1	-1.55
		P24043	LAMA2	laminin subunit alpha 2	-1.43
		P29274	ADORA2A	adenosine A2a receptor	-3.91
		P54257	HAP1	huntingtin associated protein 1	-1.38
		P56373	P2RX3	purinergic receptor P2X 3	-3.40
		Q6DN14	MCTP1	multiple C2 and transmembrane domain containing 1	-1.97
	GO:0021915 neural tube development	P15328	FOLR1	folate receptor 1	-2.20
		P50552	VASP	vasodilator stimulated phosphoprotein	-1.38
		P98161	PKD1	polycystin 1, transient receptor potential channel interacting	-2.02
		P98164	LRP2	LDL receptor related protein 2	-1.27
		Q96Q89	KIF20B	kinesin family member 20B	-1.47
		Q96RY7	IFT140	intraflagellar transport 140	-1.37

Table S3. Gene ontology of differentially expressed proteins associated with synaptic vesicle and neurotransmitter transports *(Continued)*

Time point (hpi)	GO	User ID	Gene symbol	Gene name	Fold change
72	GO:0043025 neuronal cell body	P05111	INHA	inhibin subunit alpha	0.94
		P29274	ADORA2A	adenosine A2a receptor	1.95
		P57771	RGS8	regulator of G protein signaling 8	4.76
		Q66K74	MAP1S	microtubule associated protein 1S	1.76
		Q8NER1	TRPV1	transient receptor potential cation channel subfamily V member 1	2.28
		Q92953	KCNB2	potassium voltage-gated channel subfamily B member 2	1.49
		Q9BTU6	PI4K2A	phosphatidylinositol 4-kinase type 2 alpha	2.93
	GO:0050906 detection of stimulus involved in sensory perception	Q8NER1	TRPV1	transient receptor potential cation channel subfamily V member 1	2.281
		Q8NH74	OR10A6	olfactory receptor family 10 subfamily A member 6 (gene/pseudogene)	1.178
		Q8WXG9	ADGRV1	adhesion G protein-coupled receptor V1	2.26
		Q9H5I5	PIEZO2	piezo type mechanosensitive ion channel component 2	1.69
		Q9P0L9	PKD2L1	polycystin 2 like 1, transient receptor potential cation channel	1.19

Table S4. Gene ontology of differentially expressed proteins associated with organelle and cytoskeleton organization

Time point (hpi)	GO	User ID	Gene symbol	Gene name	Fold change
	GO:0045111 intermediate filament cytoskeleton	P02545	LMNA	lamin A/C	-2.20
		P08727	KRT19	keratin 19	-1.79
		P13645	KRT10	keratin 10	-2.07
		P29274	ADORA2A	adenosine A2a receptor	-3.91
		P35527	KRT9	keratin 9	-3.02
		P48668	KRT6C	keratin 6C	-2.24
		Q86SQ0	PHLDB2	pleckstrin homology like domain family B member 2	-1.57
		Q9H307	PNN	pinin, desmosome associated protein	-2.73
	GO:0051271 negative regulation of cellular component movement	O94813	SLIT2	slit guidance ligand 2	-1.43
		P21359	NF1	neurofibromin 1	-1.55
		P35240	NF2	neurofibromin 2	-1.90
		P78563	ADARB1	adenosine deaminase, RNA specific B1	-3.11
		Q6DN14	MCTP1	multiple C2 and transmembrane domain containing 1	-1.97
		Q86SQ0	PHLDB2	pleckstrin homology like domain family B member 2	-1.57
		Q86UL8	MAGI2	membrane associated guanylate kinase, WW and PDZ domain containing 2	-2.39
		Q9NS66	GPR173	G protein-coupled receptor 173	-2.51
48	GO:0040013 negative regulation of locomotion	O94813	SLIT2	slit guidance ligand 2	-1.43
		P21359	NF1	neurofibromin 1	-1.55
		P29274	ADORA2A	adenosine A2a receptor	-3.91
		P35240	NF2	neurofibromin 2	-1.90
		P78563	ADARB1	adenosine deaminase, RNA specific B1	-3.11
		Q6DN14	MCTP1	multiple C2 and transmembrane domain containing 1	-1.97
		Q86SQ0	PHLDB2	pleckstrin homology like domain family B member 2	-1.57
		Q86UL8	MAGI2	membrane associated guanylate kinase, WW and PDZ domain containing 2	-2.39
		Q9NS66	GPR173	G protein-coupled receptor 173	-2.51
	GO:0010639 negative regulation of organelle organization	O94813	SLIT2	slit guidance ligand 2	-1.43
		P02545	LMNA	lamin A/C	-2.20
		P09327	VIL1	villin 1	-1.05
		P11277	SPTB	spectrin beta, erythrocytic	-1.77
		P16401	HIST1H1B	histone cluster 1 H1 family member b	-1.61
		P49815	TSC2	TSC complex subunit 2	-1.05
		Q7KZ85	SUPT6H	SPT6 homolog, histone chaperone	-1.15
		Q86SQ0	PHLDB2	pleckstrin homology like domain family B member 2	-1.57
		Q8NFU7	TET1	tet methylcytosine dioxygenase 1	-1.78
		Q9UBC3	DNMT3B	DNA methyltransferase 3 beta	-1.28

Table S4. Gene ontology of differentially expressed proteins associated with organelle and cytoskeleton organization ***(Continued)***

Time point (hpi)	GO	User ID	Gene symbol	Gene name	Fold change
72	GO:0031023 microtubule organizing center organization	O75116	ROCK2	Rho associated coiled-coil containing protein kinase 2	-2.15
		P53350	PLK1	polo like kinase 1	-1.34
		Q8NEM0	MCPH1	microcephalin 1	-1.31
		Q9NPB6	PARD6A	par-6 family cell polarity regulator alpha	-2.84
		Q9P2Y5	UVRAG	UV radiation resistance associated	-1.53
	GO:0045111 intermediate filament cytoskeleton	P08727	KRT19	keratin 19	1.48
		P14923	JUP	junction plakoglobin	1.94
		P29274	ADORA2A	adenosine A2a receptor	1.95
		P48668	KRT6C	keratin 6C	1.66
		Q03252	LMNB2	lamin B2	3.41
		Q92540	SMG7	SMG7, nonsense mediated mRNA decay factor	1.75

Table S5. Gene ontology of differentially expressed proteins associated with cell stress and DNA damage response

Time point (hpi)	GO	User ID	Gene symbol	Gene name	Fold change
48	GO:0071496 cellular response to external stimulus	P05023	ATP1A1	ATPase Na+/K+ transporting subunit alpha 1	-1.55
		P09529	INHBB	inhibin subunit beta B	-2.44
		P11142	HSPA8	heat shock protein family A (Hsp70) member 8	-1.45
		P15328	FOLR1	folate receptor 1	-2.35
		P56373	P2RX3	purinergic receptor P2X 3	-3.40
		Q1RMZ1	BMT2	base methyltransferase of 25S rRNA 2 homolog	-5.14
		Q8NER1	TRPV1	transient receptor potential cation channel subfamily V member 1	-1.57
		Q99836	MYD88	MYD88, innate immune signal transduction adaptor	-1.72
	GO:0042769 DNA damage response, detection of DNA damage	P35251	RFC1	replication factor C subunit 1	1.56
		P82673	MRPS35	mitochondrial ribosomal protein S35	1.60
	GO:0031072 heat shock protein binding	O75460	ERN1	endoplasmic reticulum to nucleus signaling 1	2.15
		P04150	NR3C1	nuclear receptor subfamily 3 group C member 1	1.20
		P31948	STIP1	stress induced phosphoprotein 1	2.09
72	GO:0000793 condensed chromosome	O15457	MSH4	mutS homolog 4	2.56
		O95229	ZWINT	ZW10 interacting kinetochore protein	2.62
		Q14683	SMC1A	structural maintenance of chromosomes 1A	1.41
		Q8IZU3	SYCP3	synaptonemal complex protein 3	3.13
		Q92878	RAD50	RAD50 double strand break repair protein	1.73
		Q96EA4	SPDL1	spindle apparatus coiled-coil protein 1	1.41

Table S6. Gene ontology of differentially expressed proteins associated with endocytosis

Time point (hpi)	GO	User ID	Gene symbol	Gene name	Fold change
24	GO:0005905 clathrin-coated pit	O00291	HIP1	huntingtin interacting protein 1	-3.98
		Q01968	OCRL	OCRL, inositol polyphosphate-5-phosphatase	-2.99
		Q9Y2H2	INPP5F	inositol polyphosphate-5-phosphatase F	-1.27
	GO:0031970 organelle envelope lumen	P07437	TUBB	tubulin beta class I	-1.16
		P37840	SNCA	synuclein alpha	-1.54
		Q12931	TRAP1	TNF receptor associated protein 1	-3.25
	GO:0038024 cargo receptor activity	O00291	HIP1	huntingtin interacting protein 1	-3.98
		Q5KU26	COLEC12	collectin subfamily member 12	-1.56
		Q8WTV0	SCARB1	scavenger receptor class B member 1	-1.82
		Q9UP52	TFR2	transferrin receptor 2	-2.62
	GO:0030139 endocytic vesicle	O00291	HIP1	huntingtin interacting protein 1	-3.98
		Q01968	OCRL	OCRL, inositol polyphosphate-5-phosphatase	-2.99
		Q12965	MYO1E	myosin IE	-2.64
		Q5KU26	COLEC12	collectin subfamily member 12	-1.56
		Q8WTV0	SCARB1	scavenger receptor class B member 1	-1.82
		Q9Y2H2	INPP5F	inositol polyphosphate-5-phosphatase F	-1.27
		Q9Y4G8	RAPGEF2	Rap guanine nucleotide exchange factor 2	-2.26
	GO:0006898 receptor-mediated endocytosis	O00291	HIP1	huntingtin interacting protein 1	-3.98
		P37840	SNCA	synuclein alpha	-1.54
		Q5KU26	COLEC12	collectin subfamily member 12	-1.56
		Q86UL8	MAGI2	membrane associated guanylate kinase, WW and PDZ domain containing 2	-1.64
		Q8WTV0	SCARB1	scavenger receptor class B member 1	-1.82
		Q9UP52	TFR2	transferrin receptor 2	-2.62
		Q9Y2H2	INPP5F	inositol polyphosphate-5-phosphatase F	-1.27
48	GO:0051648 vesicle localization	O14917	PCDH17	protocadherin 17	-2.35
		P12259	F5	coagulation factor V	-2.07
		P15328	FOLR1	folate receptor 1	-2.35
		P29274	ADORA2A	adenosine A2a receptor	-3.91
		P54257	HAP1	huntingtin associated protein 1	-1.38
		Q86UL8	MAGI2	membrane associated guanylate kinase, WW and PDZ domain containing 2	-2.39
		Q9BZH6	WDR11	WD repeat domain 11	-1.94

Table S6. Gene ontology of differentially expressed proteins associated with endocytosis *(continued)*

Time point (hpi)	GO	User ID	Gene symbol	Gene name	Fold change
72	GO:0005770 late endosome	P11142	HSPA8	heat shock protein family A (Hsp70) member 8	-2.14
		Q86UL8	MAGI2	membrane associated guanylate kinase, WW and PDZ domain containing 2	-1.11
		Q86VX9	MON1A	MON1 homolog A, secretory trafficking associated	-2.88
		Q8IYI6	EXOC8	exocyst complex component 8	-2.59
		Q8IZK6	MCOLN2	mucolipin 2	-2.55
		Q9P2Y5	UVRAG	UV radiation resistance associated	-1.53

Table S7. Primers for quantitative RT-PCR

Target	Forward Primer (5′-3′)	Reverse Primer (5′-3′)
CD44	CTCCAGTGAAAGGAGCAGC	AGCAGGGATTCTGTCTGTG
DCX	AAGCTTAGGTGCCTGCGTTA	AAGGGGCACTTGTGTTTGTC
GFAP	GTACCAGGACCTGCTCAAT	CAACTATCCTGCTTCTGCTC
GJA1	GTGACTGGAGCGCCTTAG	CGCACATGAGAGATTGGGA
Musashi-1	GAGACTGACGCGCCCCAGCC	CGCCTGGTCCATGAAAGTGACG
NCAM	GCCAGGAGACAGAAACGAAG	GGTGTTGGAAATGCTCTGGT
Nestin	CAGCGTTGGAACAGAGGTTGG	TGGCACAGGTGTCTCAAGGGTAG
OLIG1	GCATCCAGTGTTCCCGATTTAC	TGCCAGTTAAATTCGGCTACTAC
OLIG2	CAGAAGCGCTGATGGTCATA	TCGGCAGTTTTGGGTTATTC
PSD95	CCACTCTGACAGTGAGACC	CGTCACTGTCTCGTAGCTC
RTN1	GGGCATCGTGTTTGGGA	CTCTTAGCGCCTGGGATT
SOX2	ACACCAATCCCATCCACACT	GCAAACTTCCTGCAAAGCTC
STMN2	GCTCTTGCTTTTACCCGGAAC	ATTGTTTCAGCACCTGGGC
Syn1	GGAGAAATTGACATTAAAGTAGAACAG	CTTCTGTCCCCAGTTTCTTATGC
β-tubulin III	CAACAGCACGGCCATCCAGG	CTTGGGGCCCTGGGCCTCCGA
